# Chemosensory Gene Families in Adult Antennae of *Anomala corpulenta* Motschulsky (Coleoptera: Scarabaeidae: Rutelinae)

**DOI:** 10.1371/journal.pone.0121504

**Published:** 2015-04-09

**Authors:** Xiao Li, Qian Ju, Wencai Jie, Fei Li, Xiaojing Jiang, Jingjing Hu, Mingjing Qu

**Affiliations:** 1 Shandong Peanut Research Institute, Fushan Road 126, Licang District, Qingdao City, Shandong 266100, China; 2 Ministry of Education, Key Laboratory of Integrated Management of Crop Diseases and Pests, College of Plant Protection, Nanjing Agricultural University, Nanjing 210095, China; United States Department of Agriculture, Beltsville Agricultural Research Center, UNITED STATES

## Abstract

**Background:**

The metallic green beetle, *Anomala corpulenta* (Coleoptera: Scarabaeidae: Rutelinae), is a destructive pest in agriculture and horticulture throughout Asia, including China. Olfaction plays a crucial role in the survival and reproduction of A. corpulenta. As a non-model species, *A*. *corpulenta* is poorly understood, and information regarding the molecular mechanisms underlying olfaction in *A*. *corpulenta* and other scarab species is scant.

**Methodology/Principle Findings:**

We assembled separate antennal transcriptome for male and female *A*. *corpulenta* using Illumina sequencing technology. The relative abundance of transcripts with gene ontology annotations, including those related to olfaction in males and females was highly similar. Transcripts encoding 15 putative odorant binding proteins, five chemosensory proteins, one sensory neuron membrane protein, 43 odorant receptors, eight gustatory receptors, and five ionotropic receptors were identified. The sequences of all of these chemosensory-related transcripts were confirmed using reverse transcription polymerase chain reaction (RT-PCR), and direct DNA sequencing. The expression patterns of 54 putative chemosensory genes were analyzed using quantitative real time RT-PCR (qRT-PCR). Antenna-specific expression was detected for many of these genes, suggesting that they may have important functions in semiochemical detection.

**Conclusions:**

The identification of a large number of chemosensory proteins provides a major resource for the study of the molecular mechanism of odorant detection in *A*. *corpulenta* and its chemical ecology. The genes identified, especially those that were expressed at high levels in the antennae may represent novel molecular targets for the development of population control strategies based on the manipulation of chemoreception-driven behaviors.

## Introduction

Chemoreception plays a crucial role in many insect behaviors, such as those related to identifying suitable hosts, mates, predators, and oviposition sites [1,2]. Volatile chemical signals are detected by olfactory sensory neurons (OSNs). OSNs are primarily found on the antennal sensilla of insects and, to a lesser degree, on the maxillary palps [3]. Proteins that play crucial roles in chemoreception include odorant binding proteins (OBPs), chemosensory proteins (CSPs), sensory neuron membrane proteins (SNMPs), olfactory receptors (ORs), gustatory receptors (GRs), and ionotropic receptors (IRs) [4].

In insects, the first step in the recognition of chemical signals involves OBPs and CSPs, which are abundant in the lymph of antennal sensilla [5]. OBPs are small, water-soluble proteins composed of approximately 130 amino acids that typically contain six conserved cysteine residues [6–8]. Minus-C OBPs lack the second and fifth cysteine residues, and plus-C OBPs contain three additional conserved cysteines and one conserved proline [9]. It is presumably that OBPs act as molecular carriers of hydrophobic signaling molecules that are taken into the antennae or other sensory organs from the external environment. OBPs transport these molecules through the aqueous space, and deliver them to chemoreceptor proteins located on the membranes of OSNs [10].

CSPs belong to a smaller, more conserved protein family that is characterized by four conserved cysteines that form two disulphide bonds [11,12]. Although the molecular functions of CSPs remain poorly understood, it has been suggested that they are involved in semiochemical detection [13], and participate in some nonchemosensory functions [14,15]. Initially identified in sex-pheromone-specific OSNs of lepidopterans, SNMPs are CD36-like proteins with two transmembrane domains [16] that are thought to act as cofactors for odorant receptors in pheromone detection [17].

Insect chemoreceptors consist of three major protein families, the ORs, GRs, and IRs, whose activation generates an electrical signal that is transmitted to the brain [18,19]. The ORs are receptor proteins on the dendritic membrane surface of OSNs that are composed of approximately 400 amino acids, and contain seven transmembrane domains that form binding sites for odorant molecules [3,20,21]. Insect olfactory neurons express odorant-specific ORs and a highly conserved coreceptor, known as Orco whose ortholog in *Drosophila* is Or83b. The Orco is required for olfactory functions and the localization of specific ORs, and the Orco and specific OR form a heterodimer that acts as an ion channel [22,23]. Although GRs are members of the same receptor superfamily as ORs, GRs are primarily expressed in gustatory sensory neurons (GSNs) of gustatory organs [24]. The IRs are members of the iGluR-related olfactory receptor family that was recently identified in *Drosophila melanogaster*, and are expressed in distinct olfactory neurons that do not express ORs or GRs, suggesting that they function through molecular mechanisms that differ from those of ORs and GRs. Previous studies have shown that IRs are expressed in coeloconic OSNs, and may function in the detection of acids and ammonia [18,25,26].

Transcriptomes based on next-generation sequencing data have been used to identify chemosensory genes in species for which a complete genomic sequence databases have been unavailable. Compared to conventional homology cloning methods, which are difficult to apply to ORs because of the low levels (20%–40%) of sequence identity shared among them, these high throughput transcriptomic approaches are more efficient for large-scale gene discovery, especially with regard to the identification of highly diverse gene families [27]. Transcriptomes assembled from high-throughput sequencing data have been used to identify protein families involved in chemoreception in many lepidopterans [19,28–31], whereas such proteins have been identified in only four species of the order Coleoptera [32–34].

The scarab beetle, *Anomala corpulenta* Motschulsky, is a major insect pest in agriculture and horticulture throughout the Asian continent, including China [35]. The adults destroy the leaves of a broad range of agriculturally important host plants and the leaves and buds of various trees. The larvae of *A*. *corpulenta* damage the subterranean parts of peanuts, wheat, soybeans, and other crops, causing significant economic losses. Because of their ecological and economic importance, researchers have investigated various approaches to control *A*. *corpulenta*. However, the soil-dwelling habits of the larvae have rendered conventional chemical pesticide treatments ineffective. Chemosensory proteins in insect pests represent potential molecular targets for the development of novel population control strategies that interfere with olfaction [36]. However, relatively little is known regarding the molecular mechanisms underlying odorant detection in coleopterans.

As no genome sequence information is available for *A*. *corpulenta*, we used Illumina sequencing technology to identify olfaction-related genes in *A*. *corpulenta* by assembling antennal transcriptomes of adult males and females. We identified many chemosensory genes expressing in the antennae of *A*. *corpulenta*. Our report is the first exhaustive characterization of chemosensory multigene families of a beetle in the superfamily Scarabaeidae.

## Materials and Methods

### Insects

Live adult *A*. *corpulenta* were collected in June 2012 from fields at the Experimental Station of the Shandong Peanut Research Institute in Qingdao, China (36.75° N, 120.50° E). The insects were separated into males and females, and were reared on fresh leaves of elm tree, *Ulmus pumila* L. in rotating wire-mesh cages at 26±1°C temperature and 18% to 20% relative humidity in an environmental chamber.

### Complementary DNA (cDNA) library construction and Illumina sequencing

The antennae were severed at the base of the pedicel using forceps, and transferred immediately to microcentrifuge tubes in liquid nitrogen. The frozen antennae were stored at −80°C. Total RNA was extracted from about five hundred female or male antennae using the Trizol reagent (Invitrogen, Carlsbad, CA, USA), according to manufacturer’s protocol. The quantity and integrity of the RNA were assessed using an Agilent 2100 Bioanalyzer (Agilent Technologies, Santa Clara, CA, USA). First-strand cDNA was synthesized from 10 μg of total RNA. The cDNA libraries were constructed using reagents provided in the Illumina sequencing kit, according to the manufacturer’s recommendations, and the libraries were sequenced using an Illumina HiSeq 2000 platform at the Chinese National Human Genome Center (Shanghai, China). The *A*. *corpulenta* male and female antennal Illumina sequence data have been submitted to NCBI (accession numbers SRR1503480 and SRR1503444, respectively).

### Read assembly and sequence annotation

All sequencing reads were filtered to remove short or low-quality reads and adapter sequences using the TGI Seqclean program (http://compbio.dfci.harvard.edu/tgi/software/). We conducted the de novo assembly using the Trinity, release 25.02.2013, program [37] using the default parameters, which did not rely on mapping the sequencing reads to a reference genome. The contigs obtained were compared with the NCBI nonredundant protein database (version 31.10.2012) using the BLASTX computational tool with a threshold E-value of 1 × 10^–5^. Gene Ontology (GO) annotations were assigned using the Blast2GO program [38] through a search of the NCBI nonredundant database using the BLASTX algorithm. The E-value cut-off was set at 1 × 10^–5^. The transcripts were categorized as cellular component, molecular function, or biological process, allowing for a meta-analyses of the gene populations.

The ORFs of the annotated olfactory gene families of *A*. *corpulenta* were identified using the Getorf utility in the EMBOSS, version 6.3.1, program (http://mobyle.pasteur.fr/ cgi-bin/portal.py?#forms::getorf) [39], and verified by comparing the predicted sequences to the NCBI nonredundant protein database using the BLASTX computational tool. The cleavage sites for signal peptides were determined using the SignalP, version 4.0, program [40]. The transmembrane domains of the candidate ORs, GRs, and IRs were identified using the TMHMM, version 2.0, program [41].

### Phylogenetic analysis

The phylogenetic trees were constructed using the maximum-likelihood method based on the putative chemosensory protein sequences identified in the transcriptomes of *A*. *corpulenta*. The OBP data set contained 154 protein sequences, including 49 sequences from *Tribolium castaneum* [42], 46 sequences from *D*. *melanogaster* [43], 13 sequences from *Ips typographus* and 31 from *Dendroctonus ponderosae* [33]. The CSP data set contained 38 protein sequences, including 19, 3, 11 sequences from *T*. *castaneum*, *I*. *typographus* and *D*. *ponderosae*, respectively [33,42]. Signal peptide sequences were removed from the data set before conducting the analyses because these regions have a high substitution rate. The SNMP data set contained 11 protein sequences, including 2, 2, 3, 3 sequences from *D*. *melanogaster*, *T*. *castaneum*, *I*. *typographus* and *D*. *ponderosae*, respectively [33,44]. The OR data set contained 111 protein sequences from *T*. *castaneum* that are expressed in the head of the adult [45], 48 sequences from *Megacyllene caryae* [32], 26 sequences from *I*. *typographus* and 33 sequences from *D*. *ponderosae* (>200 aa) [33]. The final data set contained 259 sequences. The GR data set contained 68 protein sequences from *D*. *melanogaster* [46], 181 sequences from *T*. *castaneum* [47], 3 sequences from *I*. *typographus* and 2 sequences from *D*. *ponderosae* (>200 aa) [33]. The IR data set contained 137 protein sequences, including 35 sequences from *T*. *castaneum*, 79 sequences from *D*. *melanogaster* [25], 3 sequences from *I*. *typographus* and 15 sequences from *D*. *ponderosae* (>190 aa) [33].

The multiple sequence alignment was performed using the MAFFT program [48]. Alignment ambiguities and gap-containing columns were excluded from the phylogenetic analyses. The maximum-likelihood trees were constructed using the Fasttree, version 2.1.7, software [49]. The tree images were created using the iTOL web-based program [50].

### Reverse transcription and polymerase chain reaction (RT-PCR)

The sequences of all the identified chemosensory-related transcripts were verified using RT-PCR and direct DNA sequencing. Total RNA was extracted from antennae collected from male and female *A*. *corpulenta*. Primer pairs (S1 Table) were designed according to the Illumina sequencing data using the Primer Premier 5 software (Premier Biosoft, Palo Alto, CA, USA). The PCR was performed using the Titanium Taq DNA polymerase (Clontech, Mountain View, CA, USA) and olfactory-gene-specific primer pairs. Thermal cycling was performed at 94°C 5 min, followed by 30 cycles of 94°C for 50 s, 48°C to 56°C for 1 min, and 72°C for 1 min, with a final extension at 72°C for 10 min. Forty cycles of PCR were used to amplify the OR, GR, and IR sequences because of their relative low levels of abundance. The amplicons were analyzed by electrophoresis on 1.5% agarose gel, and visualized using GelRed staining (Biotium, Hayward, CA, USA). The amplicons were purified using the Qiaquick Gel Extraction Kit (Qiagen, Hilden, Germany), and one amplicon for each gene was sequenced by a commercial service provider (Invitrogen, Shanghai, China).

### Quantitative real time RT-PCR analysis

The expression of the putative chemosensory genes in different tissues was investigated using quantitative real time RT-PCR (qRT-PCR) experiments. The qRT-PCR was performed on the Bio-RAD CFX connect Real-Time System (Applied Biosystems, USA) using SYBR Green (Invitrogen, USA). Ten micrograms of total RNA sample was used for the synthesis of first-strand cDNA with reverse transcriptase (Takara, Japan) and the cDNA was then used as a template for qRT-PCR. The specific primers for qRT-PCR (S1 Table) were designed using Primer Premier 5. The reaction programs were 1–30s at 95°C, 40 cycles of 95°C for 10s and 61°C for 30s. The specificity of the PCR reactions was assessed by measuring a 65–95°C melting curve. Expression levels of the genes were calculated relative to the expression of the control gene G3PDH using the 2^-∆∆CT^ method [51]. All reactions were performed in triplicates.

## Results

### De novo assembly and gene annotation of chemosensory genes of *A*. *corpulenta*

Solexa sequencing yielded a total of 33,384,121 (99.02%) and 25,729,630 (99.09%) clean reads for the female and male transcriptomes, respectively. The clean reads from the female and male samples were assembled into 145,780 (N50 = 2,265) and 120,190 (N50 = 2,169) contigs larger than 200 bp, respectively. The largest contigs obtained from the female and male data sets were 37,531 and 41,309 bp, respectively. The assembled contigs were compared to the NCBI nonredundant protein database using BLASTX with a cut-off E-value of 1 × 10^–5^. In females, the BLASTX searches produced 92,353 hits, which comprised 63.4% of all of the contigs assembled for females. In males, the BLASTX searches produced 83,999 hits, which comprised 69.9% of all of the contigs assembled for males. The highest match percentage (44.4% in female and 44.6% in male) is to *T*. *castaneum* sequences (S2 Table). The annotated contigs that shared > 98% amino-acid sequence identity were presumed to represent the same unigene, and only the transcript with the longest ORF was included in the analysis. Based on these criteria, bioinformatics analyses identified a total of 77 unigenes from *A*. *corpulenta* transcriptomes that belonged to gene families putatively involved in insect chemoreception, including 15 OBPs, five CSPs, one SNMP, 43 ORs, eight GRs, and five IRs (Tables 1 and 2), none of which have been reported in *A*. *corpulenta* previously. The sequences of all these transcripts assembled were experimentally validated by RT-PCR and Sanger sequencing. The nucleic acid sequences of the cDNAs shared > 99% sequence identity with the corresponding sequences in the respective transcriptomes, indicating that the transcriptome assemblies were accurate. The alignment results showed that all the sequences belonging to the same chemosensory gene family had long overlapping regions without identity, which confirmed that they represented unigenes. The amino acid sequences for all these proteins are provided in S1 File.

**Table 1:** BLASTP matches for the putative OBPs, CSPs, and SNMPs of *A*. *corpulenta*.

**Gene Name**	**Acc. number**	**ORF Length(aa)**	**Complete ORF**	**Signal Peptide**	**C nb** *	**Best Blastp Match**				
						**Name**	**Acc. number**	**Species**	**E value**	**Identity (%)**
AcorOBP1	KM251641	135	Y	1–19	6	OBP1	ACX32050.2	*Holotrichia oblita*	3.00E-80	86
AcorOBP2	KM251642	96	N		3	odorant binding protein (subfamily minus-C) C04	XP_975685.1	*Tribolium castaneum*	3.00E-13	38
AcorOBP3	KM251643	142	Y	1–20	7	OBP3	ADX96030.1	*Holotrichia oblita*	4.00E-59	61
AcorOBP4	KM251644	145	Y	1–21	7	OBP4	ADX96031.1	*Holotrichia oblita*	9.00E-41	46
AcorOBP5	KM251645	149	Y	1–19	4	OBP26	AGI05179.1	*Dendroctonus ponderosae*	1.00E-10	30
AcorOBP6	KM251646	143	Y	1–19	6	OBP15	EFA12066.1	*Tribolium castaneum*	1.00E-19	36
AcorOBP7	KM251647	140	Y	1–16	6	OBP2	BAC07271.1	*Heptophylla picea*	3.00E-14	36
AcorOBP8	KM251648	144	Y	1–17	6	OBP19	EFA02960.1	*Tribolium castaneum*	3.00E-12	30
AcorOBP9	KM251649	133	Y	1–18	4	odorant binding protein (subfamily minus-C) C04	XP_975685.1	*Tribolium castaneum*	3.00E-21	39
AcorOBP10	KM251650	136	Y	1–17	6	PREDICTED: similar to putative odorant-binding protein 1	XP_975684.1	*Tribolium castaneum*	2.00E-45	60
AcorOBP11	KM251651	212	Y	1–17	12	PREDICTED: similar to odorant-binding protein	XP_972220.1	*Tribolium castaneum*	7.00E-26	28
AcorOBP12	KM251652	214	Y	1–24	8	OBP21	AGI05159.1	*Dendroctonus ponderosae*	9.00E-31	34
AcorOBP13	KM251653	223	Y	1–17	13	PREDICTED: similar to odorant-binding protein	XP_972220.1	*Tribolium castaneum*	5.00E-52	42
AcorOBP14	KM251639	134	Y	1–19	4	ASP(precursor)	NP_001106744.1	*Bombyx mori*	2.00E-14	30
AcorOBP15	KM251640	153	Y	1–20	6	PBP2	BAF79600.1	*Anomala schonfeldti*	6.00E-78	84
AcorCSP1	KM251633	136	Y	1–20	4	chemosensory protein 5 precursor	NP_001039287.1	*Tribolium castaneum*	3.00E-34	57
AcorCSP2	KM251634	108	Y	1–18	4	chemosensory protein 1 precursor	NP_001039273.1	*Tribolium castaneum*	5.00E-42	69
AcorCSP3	KM251635	130	Y	1–19	4	chemosensory protein	AFI45003.1	*Dendroctonus ponderosae*	1.00E-43	51
AcorCSP4	KM251636	261	Y	1–21	4	chemosensory protein 6 precursor	NP_001039288.1	*Tribolium castaneum*	1.00E-43	47
AcorCSP5	KM251637	126	Y	1–20	4	chemosensory protein 7 precursor	NP_001039289.1	*Tribolium castaneum*	9.00E-53	67
AcorSNMP1	KM251638	520	Y			PREDICTED: similar to sensory neuron membrane protein 1	XP_001816436.1	*Tribolium castaneum*	8.00E-140	48

* the number of conserved cysteine residues

**Table 2:** BLASTP matches for the putative ORs, GRs, and IRs of *A*. *corpulenta*.

**Gene Name**	**Acc. number**	**ORF Length(aa)**	**Complete ORF**	**TMD(NO)**	**Best Blastp Match**				
					**Name**	**Acc. number**	**Species**	**E value**	**Identity (%)**
AcorOrco	KM251654	447	Y	7	Or83b	AEG88961.1	*Holotrichia parallela*	0	90
AcorOR1	KM251655	427	Y	2	OR 167	EFA02801.1	*Tribolium castaneum*	4E-42	28
AcorOR2	KM251656	436	Y	6	OR14	CAM84012.1	*Tribolium castaneum*	1E-94	40
AcorOR3	KM251657	392	Y	7	OR37	EEZ99229.1	*Tribolium castaneum*	4E-43	28
AcorOR4	KM251658	379	Y	7	OR58	EEZ99414.1	*Tribolium castaneum*	8E-44	29
AcorOR5	KM251659	403	N	7	OR64	EFA10800.1	*Tribolium castaneum*	3E-62	33
AcorOR6	KM251660	379	Y	7	PREDICTED: similar to odorant receptor 41	XP_972901.1	*Tribolium castaneum*	1E-38	30
AcorOR7	KM251661	402	Y	8	OR14	CAM84012.1	*Tribolium castaneum*	1E-32	26
AcorOR8	KM251662	395	Y	4	OR110	EFA09299.1	*Tribolium castaneum*	1E-33	27
AcorOR9	KM251663	381	Y	7	PREDICTED: similar to odorant receptor 41	XP_972901.1	*Tribolium castaneum*	3E-48	31
AcorOR10	KM251664	416	Y	7	OR64	EFA10800.1	*Tribolium castaneum*	5E-79	34
AcorOR11	KM251665	383	Y	4	OR110	EFA09299.1	*Tribolium castaneum*	4E-30	25
AcorOR12	KM251666	441	Y	7	OR14	CAM84012.1	*Tribolium castaneum*	6E-75	38
AcorOR13	KM251667	446	Y	7	OR14	CAM84012.1	*Tribolium castaneum*	1E-79	39
AcorOR14	KM251668	411	Y	7	OR64	EFA10800.1	*Tribolium castaneum*	1E-78	36
AcorOR15	KM251669	405	Y	6	OR41	EEZ99227.1	*Tribolium castaneum*	6E-52	29
AcorOR16	KM251670	448	Y	7	OR14	CAM84012.1	*Tribolium castaneum*	9E-63	32
AcorOR17	KM251671	385	Y	5	OR67	EEZ97776.1	*Tribolium castaneum*	1E-54	31
AcorOR18	KM251672	373	Y	5	OR108	EFA09139.1	*Tribolium castaneum*	3E-31	24
AcorOR19	KM251673	376	Y	2	OR41	EEZ99227.1	*Tribolium castaneum*	2E-37	28
AcorOR20	KM251674	375	Y	6	OR61	EEZ99416.1	*Tribolium castaneum*	7E-57	33
AcorOR21	KM251675	381	Y	6	OR111	EFA09138.1	*Tribolium castaneum*	6E-27	24
AcorOR22	KM251676	410	Y	5	OR167	EFA02801.1	*Tribolium castaneum*	2E-51	29
AcorOR23	KM251677	381	Y	7	OR61	EEZ99416.1	*Tribolium castaneum*	1E-41	31
AcorOR24	KM251678	374	N	6	OR78	EFA10778.1	*Tribolium castaneum*	3E-28	24
AcorOR25	KM251679	411	Y	5	OR167	EFA02801.1	*Tribolium castaneum*	3E-37	26
AcorOR26	KM251680	413	Y	3	OR167	EFA02801.1	*Tribolium castaneum*	3E-45	30
AcorOR27	KM251681	372	N	7	OR167	EFA02801.1	*Tribolium castaneum*	5E-38	29
AcorOR28	KM251682	384	Y	7	OR59	EEZ99171.1	*Tribolium castaneum*	9E-51	31
AcorOR29	KM251683	386	Y	7	OR58	EEZ99414.1	*Tribolium castaneum*	2E-28	26
AcorOR30	KM251684	411	Y	5	OR167	EFA02801.1	*Tribolium castaneum*	3E-46	27
AcorOR31	KM251685	202	N	3	OR84	EFA10775.1	*Tribolium castaneum*	3e-26	35
AcorOR32	KM251686	184	N	2	OR112	EFA09271.1	*Tribolium castaneum*	1e-14	28
AcorOR33	KM251687	261	N	3	PREDICTED: similar to candidate odorant receptor	XP_001812261.1	*Tribolium castaneum*	2e-28	31
AcorOR34	KM251688	346	N	4	OR64	EFA10800.1	*Tribolium castaneum*	4e-42	31
AcorOR35	KM251689	223	N	2	OR108	EFA09139.1	*Tribolium castaneum*	6e-23	28
AcorOR36	KM251690	191	N	2	OR60	EEZ99415.1	*Tribolium castaneum*	8e-31	34
AcorOR37	KM251691	255	N	4	OR130	EFA02882.1	*Tribolium castaneum*	2e-13	26
AcorOR38	KM251692	256	N	4	OR127	EEZ97733.1	*Tribolium castaneum*	2e-14	27
AcorOR39	KM251693	241	N	4	OR116	EFA05716.1	*Tribolium castaneum*	1e-16	29
AcorOR40	KM251694	229	N	3	OR112	EFA09271.1	*Tribolium castaneum*	1e-21	30
AcorOR41	KM251695	408	N	6	OR167	EFA02801.1	*Tribolium castaneum*	2e-48	29
AcorOR42	KM251696	336	N	6	OR58	EEZ99414.1	*Tribolium castaneum*	5e-36	29
AcorGR1	KM251699	442	Y	6	PREDICTED: similar to Gustatory and odorant receptor 21a	XP_973273.1	*Tribolium castaneum*	0	73
AcorGR2	KM251698	437	Y	5	GR 155	EFA07633.1	*Tribolium castaneum*	1E-44	33
AcorGR3	KM251697	328	N	6	gustatory receptor candidate 24	CAL23157.2	*Tribolium castaneum*	8E-57	35
AcorGR4	KM251700	368	Y	6	PREDICTED: similar to gustatory receptor candidate 38	XP_001814661.1	*Tribolium castaneum*	8E-128	55
AcorGR5	KM251701	345	Y	7	PREDICTED: similar to trehalose receptor 1	XP_971005.2	*Tribolium castaneum*	2E-163	68
AcorGR6	KM251702	228	N	3	GR 141	EEZ97766.1	*Tribolium castaneum*	1E-12	28
AcorGR7	KM251703	103	N	2	GR 7	EFA04713.1	*Tribolium castaneum*	1E-17	41
AcorGR8	KM251704	231	N	5	PREDICTED: similar to Gustatory receptor 28b CG13788-PB	XP_968269.1	*Tribolium castaneum*	1E-12	28
AcorIR21a	KM251705	749	N	3	putative chemosensory ionotropic glutamate receptor IR21a	ADR64678.1	*Spodoptera littoralis*	0	47
AcorIR41a	KM251706	594	Y	5	putative chemosensory ionotropic receptor IR41a	ADR64681.1	*Spodoptera littoralis*	3E-95	30
AcorIR75q	KM251708	617	Y	3	PREDICTED: similar to ionotropic glutamate receptor-invertebrate	XP_968638.1	*Tribolium castaneum*	5E-175	58
AcorIR75x	KM251707	597	Y	3	PREDICTED: similar to ionotropic glutamate receptor-invertebrate	XP_966388.2	*Tribolium castaneum*	2E-102	36
AcorIRx	KM251709	845	N	3	PREDICTED: similar to ionotropic glutamate receptor subunit ia	XP_974901.2	*Tribolium castaneum*	0	49

### Gene ontology annotations

**Fig 1:**
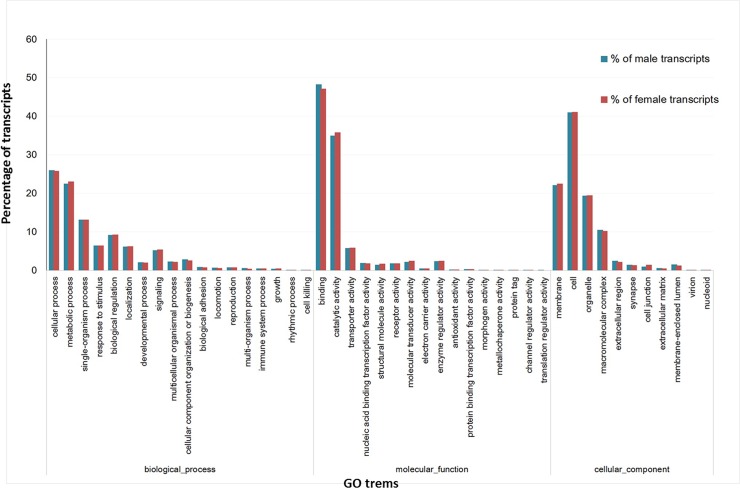
Gene ontology annotations of transcripts isolated from the antennae of *A*. *corpulenta*. The X-axis shows that transcripts were assigned to the cellular component, biological process, or molecular function categories and further to 45 subcategories at GO level 2 using the Blast2GO program. The Y-axis shows percentage of transcripts.

GO annotation indicated that the analyzed antennal transcriptomes of male and female adults were highly similar with respect to GO terms. In males, 16,702 *A*. *corpulenta* contigs were assigned to at least one GO term based on BLASTX matches with sequences with previously known function. In females, there were 18,624 such contigs. These contigs were annotated in three main categories: cellular component, biological process and molecular function, and 45 sub-categories. Among the biological process category, cellular and metabolic processes were the two largest groups in both male and female data sets. The molecular function category was mainly comprised of sequences involved in binding and catalytic activities. Under the cellular component category, cell and membrane were the most abundant GO terms (Fig 1). These GO annotations provide a global gene expression profile for male and female antenna of *A*. *corpulenta*, which is basically consistent with previously reported antenna of other Lepidoptera and Coleoptera insects [28,31,33].

### Perireceptor olfactory gene families

#### OBPs

We identified a total of 15 different sequences encoding candidate OBPs in *A*. *corpulenta*, and numbered them according to the most similar homolog whenever possible (Table 1). Of these transcripts, full-length ORFs encoding 133 to 223 amino acids were identified in 14 of them, and the remaining candidate, AcorOBP2, corresponded to a partial sequence that encoded 96 amino acids. Other unigene data for these OBPs, including length, signal peptide, closest BLASTP matches, and so on, are listed in Table 1.

Sequence analysis indicated that each of the 14 candidate OBPs that contained a complete ORF also encoded a signal peptide sequence (Table 1). The OBP1 of *A*. *corpulenta* shared 86% amino-acid sequence identity with the OBP1 of *Holotrichia oblita*, and the OBP15 of *A*. *corpulenta* shared 84% amino-acid sequence identity with the PBP2 of *Anomala schonfeldti*, whereas all other candidate OBPs of *A*. *corpulenta* displayed a low level of sequence similarity with known OBPs of coleopterans. Nine of the candidate sequences represented classic OBPs, each containing the six conserved cysteine residues (S1 Fig). AcorOBP14, AcorOBP5, and AcorOBP9 were minus-C OBPs (S1 Fig), and AcorOBP11 and AcorOBP13 were plus-C OBPs (S2 Fig) [43,52].

**Fig 2:**
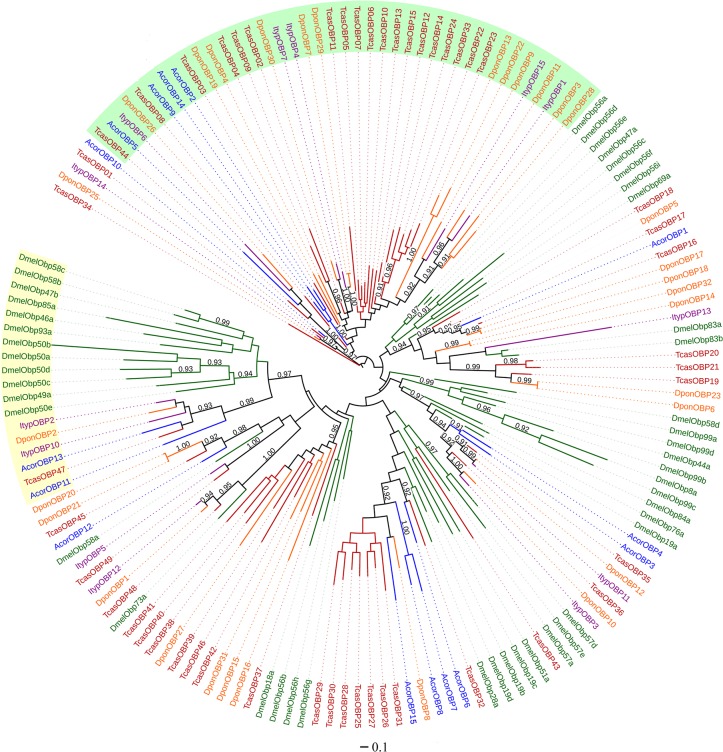
Maximum-likelihood phylogenetic tree of the putative OBPs of *A*. *corpulenta* and OBPs from *D*. *melanogaster*, *T*. *castaneum*, *I*. *typographus* and *D*. *ponderosae*. The tree was constructed using the FastTree 2.1.7 program. The numbers above the nodes indicate support values. Support values < 0.9 are not shown. The plus-C subfamily is marked in light yellow, and the minus-C subfamily is marked in light green.

To confirm the annotation and identify potential orthologs, a maximum-likelihood cluster analysis was performed (Fig 2). The phylogenetic tree had relatively large minus-C OBP and plus-C OBP branches. The OBPs of *A*. *corpulenta* were distributed over various branches, with all forming small clusters with the OBPs of coleopteran species. Of the 15 putative OBPs, seven clustered with other OBPs of *A*. *corpulenta*, rather than those of other insect species, suggesting that they may be the products of recent gene duplications.

#### CSPs

**Fig 3:**
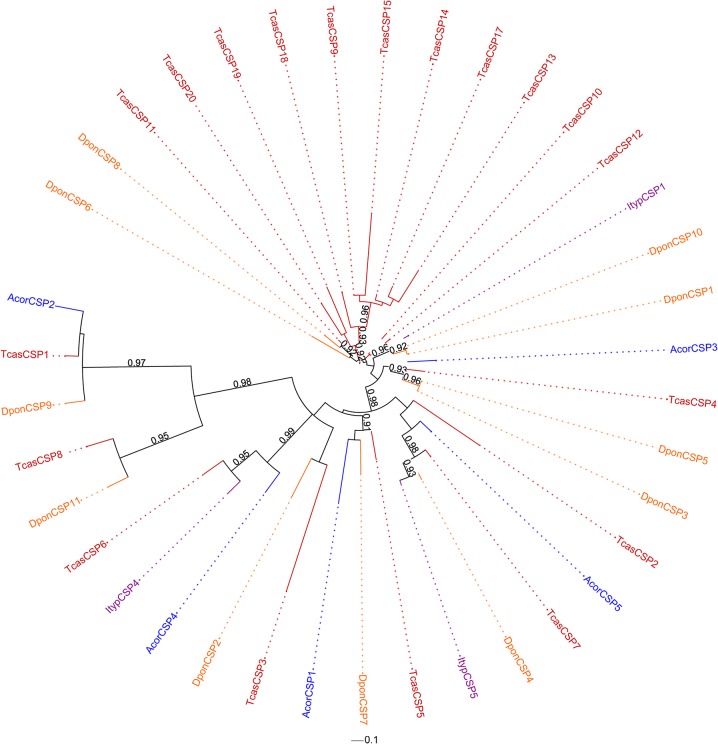
Maximum-likelihood phylogenetic tree of the putative CSPs of *A*. *corpulenta* and CSPs from *T*. *castaneum*, *I*. *typographus* and *D*. *ponderosae*. The tree was constructed using the FastTree 2.1.7 program. The numbers above the nodes indicate support values. Support values < 0.9 are not shown.

Our bioinformatic analysis identified five unigenes encoding proteins that were 108 to 261 amino acids in length, which is consistent with other insect CSPs (Table 1). Each of the putative CSP unigenes of *A*. *corpulenta* had an intact ORF with a signal peptide and four conserved cysteine residues. The maximum-likelihood tree showed that AcorCSP3 clustered confidently with TcasCSP4, DponCSP3 and DponCSP5. AcorCSP4 clustered confidently with TcasCSP6 and ItypCSP4. There were weak values supporting the other three clades: AcorCSP1/DponCSP7, AcorCSP2/TcasCSP1 and AcorCSP5/TcasCSP7/DponCSP4/ItypCSP5 (Fig 3).

#### SNMPs

In general, SNMPs are classified as SNMP1 or SNMP2. Based on the transcriptome data, only one SNMP candidate was identified in the antennae of *A*. *corpulenta*. Phylogenetic analysis along with BLASTP search revealed it was classified as SNMP1 (Table 1, S3 Fig). The AcorSNMP1 candidate shared 48% amino-acid sequence identity with an SNMP1 in *T*. *castaneum* (XP_001816436.1; Table 1).

### Receptor encoding genes families

#### ORs

Forty-three unigenes encoding putative ORs were identified based on the antennal transcriptome data for *A*. *corpulenta*. Transcripts for all the candidate ORs were present in the transcriptomes of both sexes. Among them, 28 contained a full-length ORF that encoded 372 to 448 amino acids. Fifteen partial sequences encoded 184–408 amino acids. One OR sequence was found that shared a high level of identity with the conserved Orco proteins of other insect species and named as AcorOrco. The amino-acid sequence of AcorOrco shared 90% identity with Or83b of *Holotrichia parallela* (AEG88961.1) and 81% identity with the Orco of *T*. *castaneum* (XP_008194693.1). The other ORs of *A*. *corpulenta* were highly divergent, and shared low levels of similarity with other insect ORs. Between two and eight transmembrane domains were identified in the ORs of *A*. *corpulenta* (Table 2), based on comparisons with other insect ORs [19]. Thirteen (46.4%) of the 28 ORs with complete ORF of *A*. *corpulenta* contained seven transmembrane domains, which is characteristic of the OR protein family. The topology analysis indicated that the N-terminus of 21 of the 28 full-length ORs of *A*. *corpulenta* was on the cytoplasmic side of the membrane. This is the typical membrane topology of insect ORs and is inverted relative to vertebrate ORs [21].

**Fig 4:**
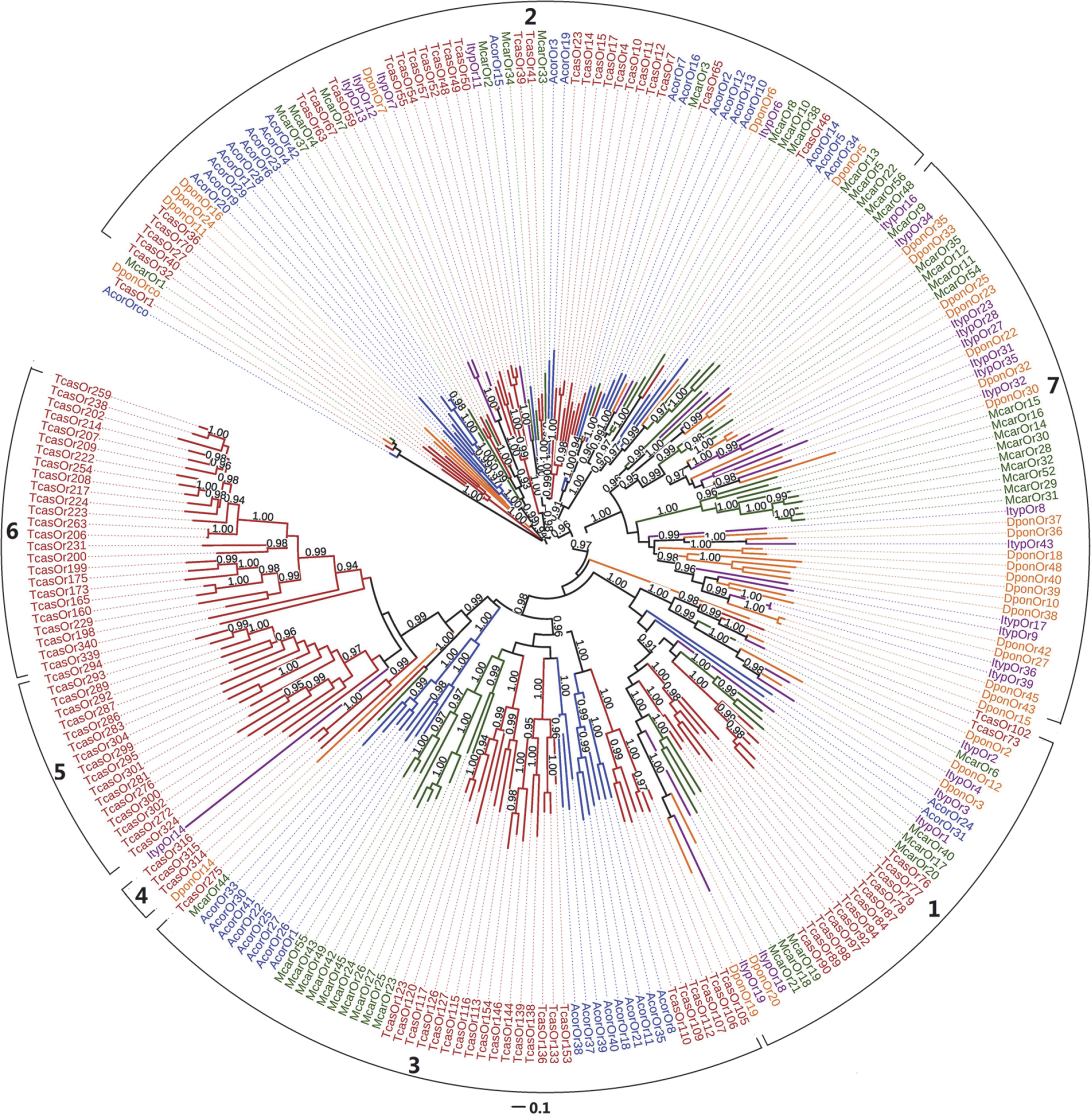
Maximum-likelihood tree of the putative ORs of *A*. *corpulenta* and ORs of *T*. *castaneum*, *M*. *caryae*, *I*. *typographus* and *D*. *ponderosae*. The tree was constructed using the FastTree 2.1.7 program. The numbers above the nodes indicate support values. Support values < 0.9 are not shown.

A maximum-likelihood phylogenetic analysis was performed using a data set containing the sequences of the ORs that were longer than 200 amino acids from *A*. *corpulenta* and other four described coleopteran species [32,33,45] (Fig 4). The OR sequences were clustered into several subgroups which were numbered from 1 to 7 according to previous studies [32,33,45]. Except for AcorOrco, *A*. *corpulenta* ORs were only present within previously defined coleopteran OR subgroup1, 2, and 3. The majority of the ORs of *A*. *corpulenta* clustered into species-specific expansions within receptor subgroups 2 and 3. As previously reported, receptor subgroups 4, 5, and 6 contained ORs from *T*. *castaneum*, and subgroup 7 contained ORs from *M*. *caryae* only [32,33,45], with one exception, ItypOR14, which was clustered within subgroup 5 but showed a weak supporting value. The orthologs to the functionally characterized ORs of *M*. *caryae* were identified in *A*. *corpulenta*. McarOR3 and TcasOR65 formed a strong lineage with AcorOR2, AcorOR12, and AcorOR13. AcorOR34 was clustered confidently with DponOR5 and five *M*. *caryae* olfactory receptors including McarOR5. However, McarOR20 represented no apparent orthologs to any ORs of *A*. *corpulenta*.

#### GRs

Bioinformatic analysis identified eight putative GR unigenes. Among them, a complete ORF was identified in AcorGR2, AcorGR3, AcorGR4, and AcorGR5, and the remaining GRs were partial sequences that encoded more than 103 amino acids. Based on the phylogenetic analysis, several potential orthologs for other known insect GRs were identified. AcorGR2 represented an ortholog with TcasGR155. AcorGR5 clustered confidently with ItypGR6. Besides, two *A*. *corpulenta* GRs clustered within the highly conserved lineage of the DmelGR21a and DmelGR63a. AcorGR3 represented an ortholog to TcasGR1, DponGR1 and DmelGR63a. AcorGR4 was the orthologous receptor of TcasGR3, ItypGR3, DponGR3 and DmelGR63a (S4 Fig).

#### IRs

**Fig 5:**
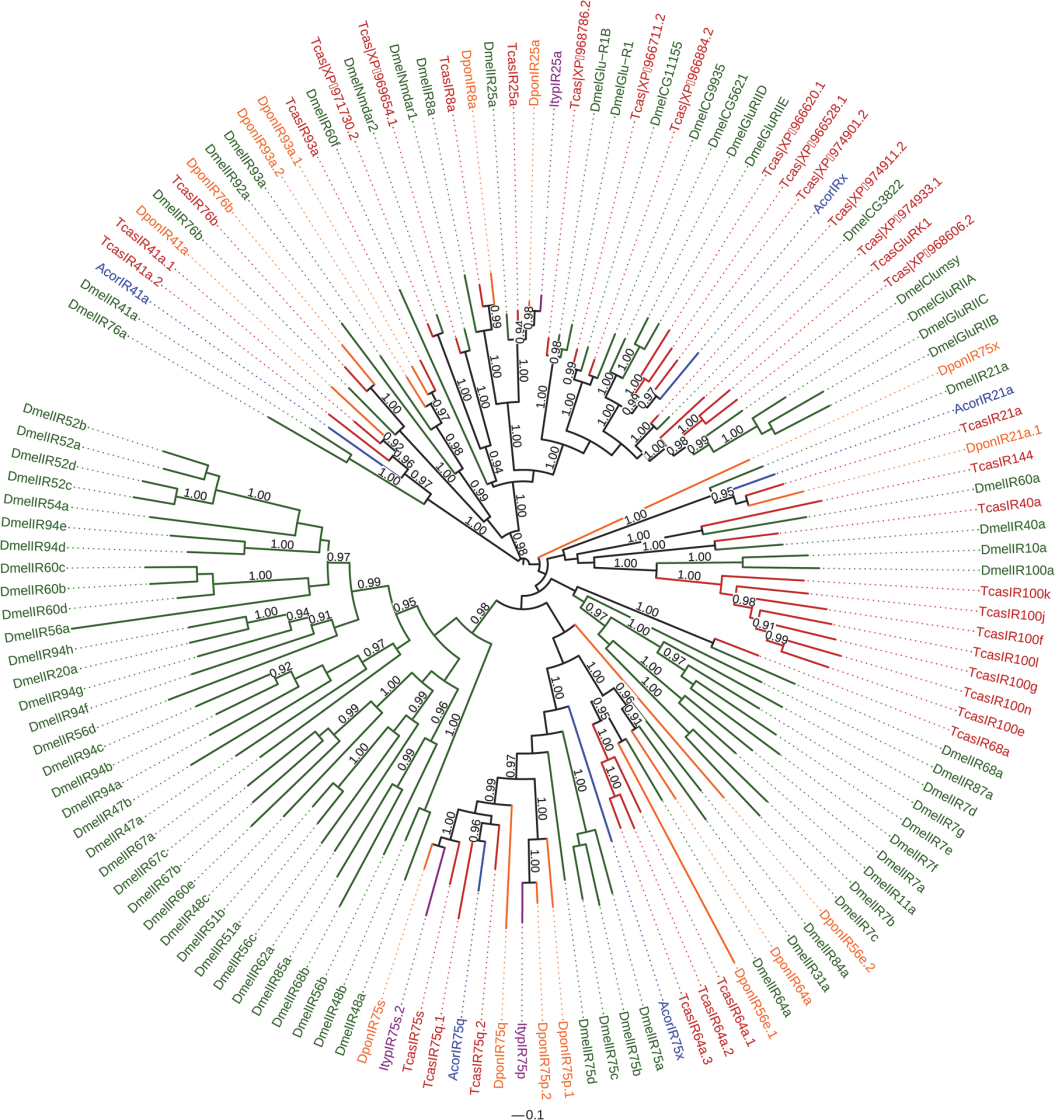
Maximum-likelihood tree of the putative IRs of *A*. *corpulenta* and IRs of *D*. *melanogaster*, *T*. *castaneum*, *I*. *typographus* and *D*. *ponderosae*. The tree was constructed using the FastTree 2.1.7 program. The numbers above the nodes indicate support values. Support values < 0.9 are not shown.

We identified five putative IR unigenes in *A*. *corpulenta*, in three of which a complete ORF was identified. Insect IRs typically contain three transmembrane domains [25,26]. We identified three transmembrane domains in four of the IRs of *A*. *corpulenta* (Table 2). According to the maximum-likelihood tree of the IRs from *D*. *melanogaster* and four coleopterans, we found each member of IR21a, IR41a and IR75q lineage in *A*. *corpulenta* (Fig 5). However, we didn’t find the ortholog for co-receptor IR8a or IR25a. One *A*. *corpulenta* IR located at the base of a branch formed by 14 homologs belonging to IR75 group but its exact orthologues were not identified (Fig 5). Thus it was named AcorIR75x. Besides, one *A*. *corpulenta* IR which clustered with an IR (XP_974901.2) from *T*. *castaneum* was named AcorIRx (Fig 5).

### Quantitative real time RT-PCR analysis

**Fig 6:**
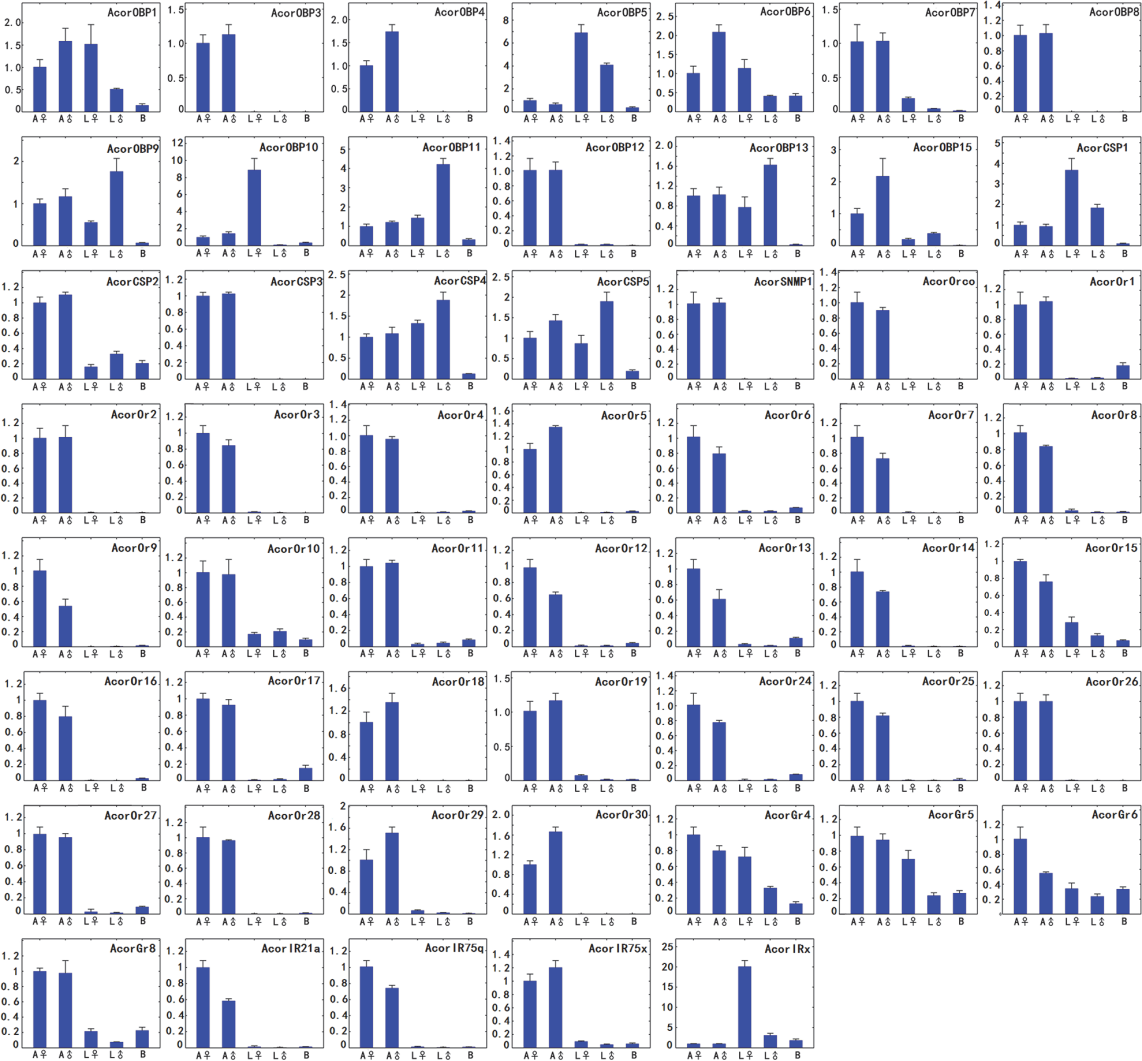
Relative expression levels of the putative chemosensory transcripts of *A*. *corpulenta* using qRT-PCR. The X-axis shows the different body parts. The Y-axis shows the relative mRNA level. A♀, female antennae; A♂, male antennae; L♀, female legs; L♂, male, legs; B, body tissues (mixture of male thorax and abdomen).

We investigated the expression patterns of 54 putative *A*. *corpulenta* chemosensory genes by performing a qRT-PCR analysis of the RNA extracted from adult male and female antennae, male and female legs as well as non-chemosensory tissues (mixture of male thorax and abdomen which was referred to as body tissues here after) (Fig 6). Among the 13 tested OBPs, AcorOBP3, AcorOBP4, AcorOBP7, AcorOBP8 and AcorOBP12 displayed antenna-specific expression without sex bias except AcorOBP4, which was expressed at a higher level in male than in female. The remaining OBPs were expressed in the legs and non-chemosensory tissues as well as in the antennae. Of these widely distributed genes, AcorOBP1, AcorOBP6 and AcorOBP15 were expressed at the highest level in male antennae compared with other body parts. The analysis on the expression of all the five *A*. *corpulenta* CSPs was performed. AcorCSP3 displayed a clear antennae-specific expression with approximately equivalent levels in both sexes. The remaining four CSPs were found to be expressed not only in antennae but also in legs and no-chemosensory tissues. The SNMP1 of *A*. *corpulenta* was expressed only in the antennae with no significant difference observed in the level between males and females.

The majority of the tested ORs were clearly antenna-specific. AcorOR10 and AcorOR15 were expressed in legs at lower levels relative to in the antennae. AcorOrco was only expressed in the antennae with no sex bias. Although we didn't find apparent sex-specific genes in these *A*. *corpulenta* olfactory receptors, AcorOR7, AcorOR9, AcorOR12, AcorOR13, AcorOR15 and AcorOR25 were more highly expressed in male antennae than in female antennae, wheras AcorOR5, AcorOR29 and AcorOR30 were expressed at higher levels in male antennae than in female antennae. All the four tested GRs were not only expressed in the antennae but also in the legs and the body tissues. AcorGR4 and AcorGR6 were more highly expressed in female antennae than in male antenna. Of the four tested IRs, AcorIR21a, AcorIR75q and AcorIR75x displayed an antenna-specific expression, among which AcorIR21a and AcorIR75q were expressed at a higher level in female than in male. AcorIRx was expressed more highly in the legs compared with in the antennae and body tissues.

## Discussion

The molecular basis of chemoreception in coleopterans is relatively poorly understood compared to dipterans and lepidopterans. Major chemosensory gene families have been identified in the red flour beetle, *T*. *castaneum*, and three species of bark beetles [32–34]. We generated antennal transcriptomes of male and female *A*. *corpulenta* using Illumina next-generation sequencing technology. We identified 77 chemosensory genes encoding members of six protein families, including OBPs, CSPs, SNMPs, ORs, GRs, and IRs. However, transcripts present at low abundance and those that are too divergent to be identified using a BLAST search may be overlooked in transcriptome analysis. Thus, it is unlikely that the identified genes represent the total number of the related chemosensory genes *in A*.*corpulenta*. This is the first comprehensive characterization of olfactory genes in the scarab beetle. Our findings provide insight into the molecular mechanisms of olfaction in *A*. *corpulenta*, and identify potential molecular targets for controlling this pest.

A total of 15 OBPs were identified in the antennal transcriptome of *A*. *corpulenta*. This number is equal to that of OBPs previously identified in the antennae of *I*. *typographus*. By contrast, more OBPs were identified in *T*. *castaneum* based on a genomic analysis, and more were identified in *D*. *ponderosae* based on the transcriptome analysis of various tissues, including the antenna, from different life stages (S3 Table). Our analysis of the antennal transcriptome may not have identified OBPs expressed primarily or exclusively in other tissues or life stages, such as in larve. OBPs may share a common ancestor with CSPs [42]. The amino-acid sequence similarity between the OBPs and CSPs of *A*. *corpulenta* and those of other insect species indicates that OBPs are less conserved than the CSPs [42]. The antennae-specific expression of OBPs has been implicated in pheromone detection, whereas expression of OBPs in other organs has been shown to be related to the other functions apart from chemosensation [36]., Some OBPs exhibited antenna-specific pattern of expression, suggesting they may play important roles in detecting odor molecules. Future functional investigation of these genes is warranted to determine their specific roles in chemical communication.

CSPs constitute another class of small binding proteins. Although most of the genomes of *Drosophila* species contain only four CSP genes, *T*. *castaneum*, which is more closely related to *A*. *corpulenta*, has 20 CSPs [42]. We identified a modest number of CSPs in *A*. *corpulenta*, presumably because the expression of many CSP genes is limited to nonsensory tissues or other developmental stages [53]. Among the five predicted CSPs of *A*. *corpulenta*, four were detected in both the antennae and non-chemosensory tissues, which is consistent with the expression patterns of CSPs in other insects [54,55]. Thus, in addition to chemosensation function, CSPs in *A*.*corpulenta* may be involved in other functions such as the regeneration of legs [15] and female survival and reproduction [56]. AcorCSP3 displayed apparent antenna-specific expression suggesting that it may play an important role in semiochemical recognition.

SNMPs can generally be categorized as SNMP1 or SNMP2, according to sequence similarity and intron locations [44]. Although the functions of SNMPs remain largely unclear, SNMPs have been shown to be essential for pheromone responses [16]. We identified only one SNMP in the antennal transcriptome of *A*. *corpulenta*, which we classified as an SNMP1, and found that it clearly displayed antenna-specific expression in both sexes, suggesting it may be important in chemoreception. The SNMPs are conserved throughout holometabolous insects [57]. In certain moths, a high level of antenna-specific expression has been observed for SNMP1, whereas SNMP2 is expressed at various levels in a variety of tissues [57,58]. However, studies of SNMPs in coleopterans are scant. We speculate that the tissue specificity of AcorSNMP2 expression may differ from that reported in other coleopterans [33]. In addition, an SNMP2 ortholog was not found in an analysis of another scarab chafer, *H*. *parallela* (unpublished). Therefore, we speculate that the level of SNMP2 expression in the antenna of *A*. *corpulenta* may be low, and thus undetectable based on the transcriptome analysis. It is also possible that members of Scarabaeidae may have lost SNMP2 during evolution.

ORs of coleopterans from the superfamilies Tenebrionoidea, Chrysomeloidea, and Curculionidae have been described, and the findings of our study extend the knowledge base of coleopteran ORs to include the superfamily Scarabaeidae. In insects, ORs have undergone frequent gene duplications and deletion events, representing the “birth-to-death” model of evolution, which has contributed to a high level of diversity in OR genes and variability in gene number between different species. Sixty-three ORs have been identified in *D*. *melanogaster* [46], and hundreds of ORs have been identified in ants [59]. Relatively few ORs were identified in the antennal transcriptomes of *A*. *corpulenta*, compared with the number of ORs in the complete genome of *T*. *castaneum*, but comparable to the number of 57 ORs identified in the antennal transcriptome of *M*. *caryae*. Maybe lower expressed ORs were not detected. However, similar to observations in *T*. *castaneum* and *M*. *caryae*, a species-specific expansion of ORs was observed in *A*. *corpulenta*. These species-specific expansions may reflect the different ecological niches of these species. ORs play a key role in odor specification. We may speculate on the possible functions of ORs in *A*. *corpulenta* through those of orthologous ORs in *M*. *caryae*, which was the first beetle in which the function of ORs was characterized. However, the effects of the rapid evolution of OR families might be inconsistent with such speculation. The AcorOR2, AcorOR12, and AcorOR13 clustered into a clade that was a sister group of McarOR3/TcasOR65. McarOR3 binds (*S*)-2-methyl-1-butanol and structurally related chemicals. Whether AcorOR2, AcorOR12, and AcorOR13 have functions that are similar to those of McarOR3 warrants further investigation [32]. The expression profiling indicated that *A*. *corpulenta* ORs were highly restricted in the antennae relative to OBPs and CSPs. We also found several ORs expressed in legs and no-chemosensory tissues, similar to the findings in *Sesamia inferens*[60]. Unlike *T*. *castaneum* olfactory co-receptor which was also expressed in legs and other tissues [61], AcorOrco showed a clear antennae-specific expression pattern.

A large number of GRs have also been identified in various insect species [24,46,62]. We identified a small number of GRs in *A*. *corpulenta*, which was expected because GRs are primarily expressed in gustatory organs, such as the proboscis, rather than the antennae [24]. A previous study showed that GR21a and GR63a of *D*. *melanogaster* form heterodimers, and are involved in CO_2_ detection [63]. Both orthologs of these two receptors were found in the antenna transcriptomes of *A*. *corpulenta*, which suggests that *A*. *corpulenta* might use CO_2_ detection as a criterion for host plant selection. In contrast to AcorORs, AcorGRs were widely expressed in different tissues with relatively high leg expression, similar to that reported in *T*. *castaneum* [61].

Compared with the ORs and GRs, IRs are more highly conserved in insects, and the number of IRs is generally consistent [25]. IRs can be categorized as divergent species-specific IRs and conserved antennal IRs, the latter of which are expressed in the sensory dendrites of antennae [18,25]. For example, of the 66 IRs identified in drosophilids, 15 were antennal IRs [18]. The orthologs of IR21a, IR41a, and IR75q of *T*. *castaneum* were identified in *A*. *corpulenta*. AcorIR21a, AcorIR75q and AcorIR75x displayed antenna-specific expression. Our findings suggest that these IRs may function in olfaction in *A*. *corpulenta*.

This study not only described the putative *A*.*corpulenta* chemosensory genes identified from the antennal transcriptome, but also investigated the expression profile of these genes. The genes that are potentially involved in *A*. *corpulenta* chemical communication may represent novel molecular targets for controlling this beetle by regulating chemoreception.

## Supporting Information

S1 Fig Amino-acid sequence alignment of the putative classic OBPs and minus-C OBPs of *A*. *corpulenta* with OBPs of *D*. *melanogaster*. The alignment was conducted using the ClustalW 2.0 program.
(TIF)

S2 Fig Amino-acid sequence alignment of the putative plus-C OBPs of *A*. *corpulenta* with OBPs of *D*. *melanogaster*. The alignment was conducted using the ClustalW 2.0 program.
(TIF)

S3 Fig Maximum-likelihood tree of the putative SNMP1 of *A*. *corpulenta* and SNMPs of *D*. *melanogaster*, *T*. *castaneum*, *I*. *typographus* and *D*. *ponderosae*. The tree was constructed using the FastTree 2.1.7 program. The numbers above the nodes indicate support values. Support values < 0.9 are not shown.
(TIF)

S4 Fig Maximum-likelihood tree of the putative GRs of *A*. *corpulenta* and GRs of *T*. *castaneum*, *I*. *typographus* and *D*. *ponderosae*. The tree was constructed using the FastTree 2.1.7 program. The numbers above the nodes indicate support values. Support values < 0.9 are not shown.
(TIF)

S1 File FASTA format of the amino acid sequences of the *A*. *Corpulenta* chemosensory genes. (PDF)

S1 Table PCR primers used for RT-PCR and qRT-PCR. (PDF)

S2 Table Percentage of homologous hits of the *A*. *corpulenta* contigs to other insect species. (PDF)

S3 Table Comparison of the number of chemosensory genes identified in different coleopteran species. (PDF)
