# Correction: Chemosensory Gene Families in Adult Antennae of *Anomala corpulenta* Motschulsky (Coleoptera: Scarabaeidae: Rutelinae)

**DOI:** 10.1371/journal.pone.0144214

**Published:** 2015-11-30

**Authors:** Xiao Li, Qian Ju, Wencai Jie, Fei Li, Xiaojing Jiang, Jingjing Hu, Mingjing Qu

There is an error in the final sentence of the GRs sub-subsection of the Receptor encoding genes families subsection within the Results section. The value DmelGR63a should be DmelGR21a. The correct sentence is as follows: AcorGR3 represented an ortholog to TcasGR1, DponGR1 and DmelGR63a. AcorGR4 was the orthologous receptor of TcasGR3, ItypGR3, DponGR3 and DmelGR21a (S4 Fig).

Additionally, in Table 2, the values AcorGR1 and AcorGR3 were swapped in the first column. Please see the corrected Table 2 here.

**Table 2:** BLASTP matches for the putative ORs, GRs, and IRs of *A*. *corpulenta*.

Gene Name	Acc. number	ORF Length(aa)	Complete ORF	TMD (NO)	Best Blastp Match				
					Name	Acc. number	Species	E value	Identity (%)
AcorOrco	KM251654	447	Y	7	Or83b	AEG88961.1	*Holotrichia parallela*	0	90
AcorOR1	KM251655	427	Y	2	OR 167	EFA02801.1	*Tribolium castaneum*	4E-42	28
AcorOR2	KM251656	436	Y	6	OR14	CAM84012.1	*Tribolium castaneum*	1E-94	40
AcorOR3	KM251657	392	Y	7	OR37	EEZ99229.1	*Tribolium castaneum*	4E-43	28
AcorOR4	KM251658	379	Y	7	OR58	EEZ99414.1	*Tribolium castaneum*	8E-44	29
AcorOR5	KM251659	403	N	7	OR64	EFA10800.1	*Tribolium castaneum*	3E-62	33
AcorOR6	KM251660	379	Y	7	PREDICTED: similar to odorant receptor 41	XP_972901.1	*Tribolium castaneum*	1E-38	30
AcorOR7	KM251661	402	Y	8	OR14	CAM84012.1	*Tribolium castaneum*	1E-32	26
AcorOR8	KM251662	395	Y	4	OR110	EFA09299.1	*Tribolium castaneum*	1E-33	27
AcorOR9	KM251663	381	Y	7	PREDICTED: similar to odorant receptor 41	XP_972901.1	*Tribolium castaneum*	3E-48	31
AcorOR10	KM251664	416	Y	7	OR64	EFA10800.1	*Tribolium castaneum*	5E-79	34
AcorOR11	KM251665	383	Y	4	OR110	EFA09299.1	*Tribolium castaneum*	4E-30	25
AcorOR12	KM251666	441	Y	7	OR14	CAM84012.1	*Tribolium castaneum*	6E-75	38
AcorOR13	KM251667	446	Y	7	OR14	CAM84012.1	*Tribolium castaneum*	1E-79	39
AcorOR14	KM251668	411	Y	7	OR64	EFA10800.1	*Tribolium castaneum*	1E-78	36
AcorOR15	KM251669	405	Y	6	OR41	EEZ99227.1	*Tribolium castaneum*	6E-52	29
AcorOR16	KM251670	448	Y	7	OR14	CAM84012.1	*Tribolium castaneum*	9E-63	32
AcorOR17	KM251671	385	Y	5	OR67	EEZ97776.1	*Tribolium castaneum*	1E-54	31
AcorOR18	KM251672	373	Y	5	OR108	EFA09139.1	*Tribolium castaneum*	3E-31	24
AcorOR19	KM251673	376	Y	2	OR41	EEZ99227.1	*Tribolium castaneum*	2E-37	28
AcorOR20	KM251674	375	Y	6	OR61	EEZ99416.1	*Tribolium castaneum*	7E-57	33
AcorOR21	KM251675	381	Y	6	OR111	EFA09138.1	*Tribolium castaneum*	6E-27	24
AcorOR22	KM251676	410	Y	5	OR167	EFA02801.1	*Tribolium castaneum*	2E-51	29
AcorOR23	KM251677	381	Y	7	OR61	EEZ99416.1	*Tribolium castaneum*	1E-41	31
AcorOR24	KM251678	374	N	6	OR78	EFA10778.1	*Tribolium castaneum*	3E-28	24
AcorOR25	KM251679	411	Y	5	OR167	EFA02801.1	*Tribolium castaneum*	3E-37	26
AcorOR26	KM251680	413	Y	3	OR167	EFA02801.1	*Tribolium castaneum*	3E-45	30
AcorOR27	KM251681	372	N	7	OR167	EFA02801.1	*Tribolium castaneum*	5E-38	29
AcorOR28	KM251682	384	Y	7	OR59	EEZ99171.1	*Tribolium castaneum*	9E-51	31
AcorOR29	KM251683	386	Y	7	OR58	EEZ99414.1	*Tribolium castaneum*	2E-28	26
AcorOR30	KM251684	411	Y	5	OR167	EFA02801.1	*Tribolium castaneum*	3E-46	27
AcorOR31	KM251685	202	N	3	OR84	EFA10775.1	*Tribolium castaneum*	3e-26	35
AcorOR32	KM251686	184	N	2	OR112	EFA09271.1	*Tribolium castaneum*	1e-14	28
AcorOR33	KM251687	261	N	3	PREDICTED: similar to candidate odorant receptor	XP_001812261.1	*Tribolium castaneum*	2e-28	31
AcorOR34	KM251688	346	N	4	OR64	EFA10800.1	*Tribolium castaneum*	4e-42	31
AcorOR35	KM251689	223	N	2	OR108	EFA09139.1	*Tribolium castaneum*	6e-23	28
AcorOR36	KM251690	191	N	2	OR60	EEZ99415.1	*Tribolium castaneum*	8e-31	34
AcorOR37	KM251691	255	N	4	OR130	EFA02882.1	*Tribolium castaneum*	2e-13	26
AcorOR38	KM251692	256	N	4	OR127	EEZ97733.1	*Tribolium castaneum*	2e-14	27
AcorOR39	KM251693	241	N	4	OR116	EFA05716.1	*Tribolium castaneum*	1e-16	29
AcorOR40	KM251694	229	N	3	OR112	EFA09271.1	*Tribolium castaneum*	1e-21	30
AcorOR41	KM251695	408	N	6	OR167	EFA02801.1	*Tribolium castaneum*	2e-48	29
AcorOR42	KM251696	336	N	6	OR58	EEZ99414.1	*Tribolium castaneum*	5e-36	29
AcorGR3	KM251699	442	Y	6	PREDICTED: similar to Gustatory and odorant receptor 21a	XP_973273.1	*Tribolium castaneum*	0	73
AcorGR2	KM251698	437	Y	5	GR 155	EFA07633.1	*Tribolium castaneum*	1E-44	33
AcorGR1	KM251697	328	N	6	gustatory receptor candidate 24	CAL23157.2	*Tribolium castaneum*	8E-57	35
AcorGR4	KM251700	368	Y	6	PREDICTED: similar to gustatory receptor candidate 38	XP_001814661.1	*Tribolium castaneum*	8E-128	55
AcorGR5	KM251701	345	Y	7	PREDICTED: similar to trehalose receptor 1	XP_971005.2	*Tribolium castaneum*	2E-163	68
AcorGR6	KM251702	228	N	3	GR 141	EEZ97766.1	*Tribolium castaneum*	1E-12	28
AcorGR7	KM251703	103	N	2	GR 7	EFA04713.1	*Tribolium castaneum*	1E-17	41
AcorGR8	KM251704	231	N	5	PREDICTED: similar to Gustatory receptor 28b CG13788-PB	XP_968269.1	*Tribolium castaneum*	1E-12	28
AcorIR21a	KM251705	749	N	3	putative chemosensory ionotropic glutamate receptor IR21a	ADR64678.1	*Spodoptera littoralis*	0	47
AcorIR41a	KM251706	594	Y	5	putative chemosensory ionotropic receptor IR41a	ADR64681.1	*Spodoptera littoralis*	3E-95	30
AcorIR75q	KM251708	617	Y	3	PREDICTED: similar to ionotropic glutamate receptor-invertebrate	XP_968638.1	*Tribolium castaneum*	5E-175	58
AcorIR75x	KM251707	597	Y	3	PREDICTED: similar to ionotropic glutamate receptor-invertebrate	XP_966388.2	*Tribolium castaneum*	2E-102	36
AcorIRx	KM251709	845	N	3	PREDICTED: similar to ionotropic glutamate receptor subunit ia	XP_974901.2	*Tribolium castaneum*	0	49
