# Supplementary figures and images for: Chemosensory Gene Families in Adult Antennae of Anomala corpulenta Motschulsky (Coleoptera: Scarabaeidae: Rutelinae)
Source: PLoS One. 2015 Apr 9;10(4):e0121504. doi: 10.1371/journal.pone.0121504 (PMC4391716; doi:10.1371/journal.pone.0121504)

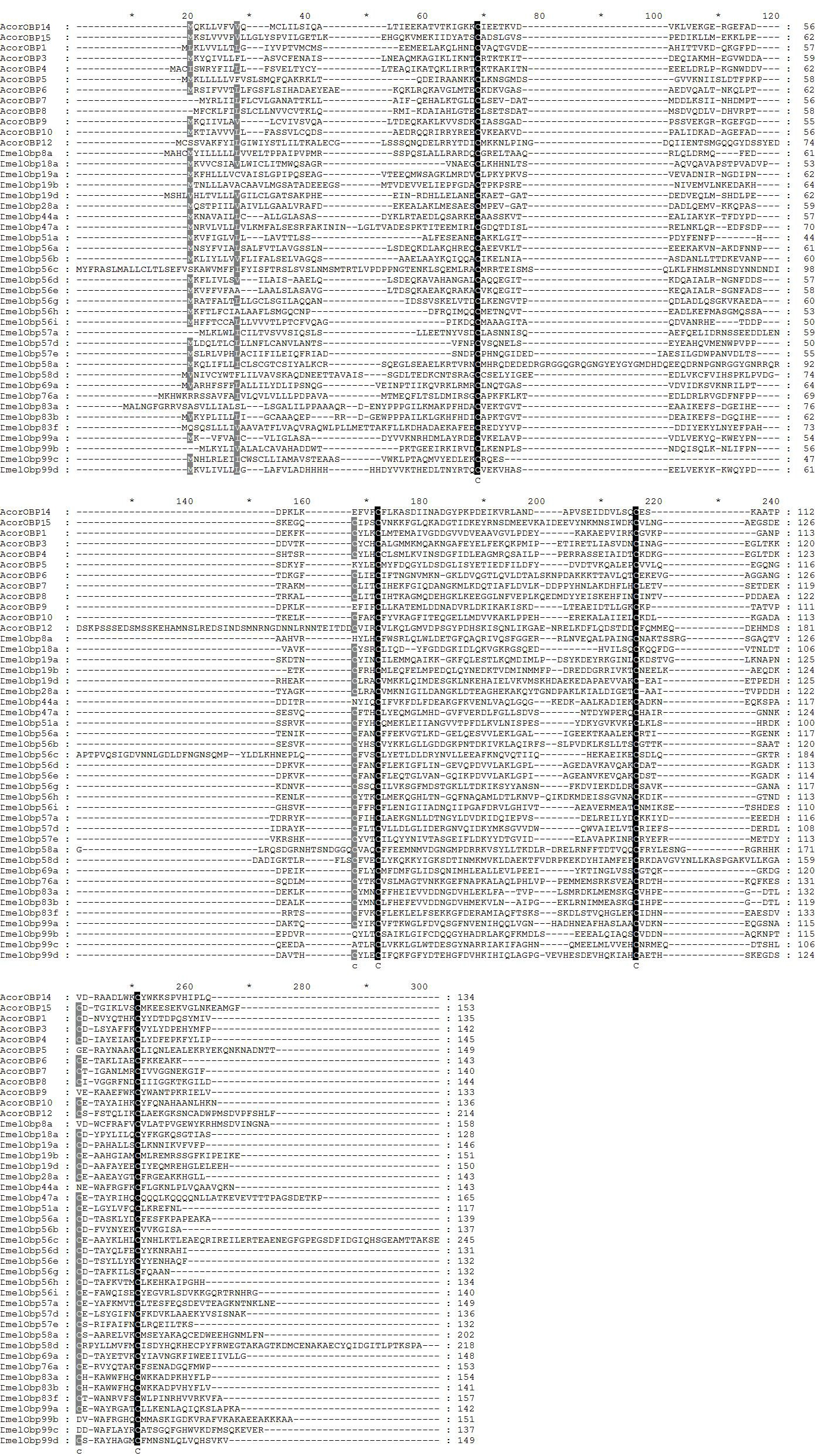

Supplement: S1 Fig — The alignment was conducted using the ClustalW 2.0 program. (TIF) [file pone.0121504.s001.tif]

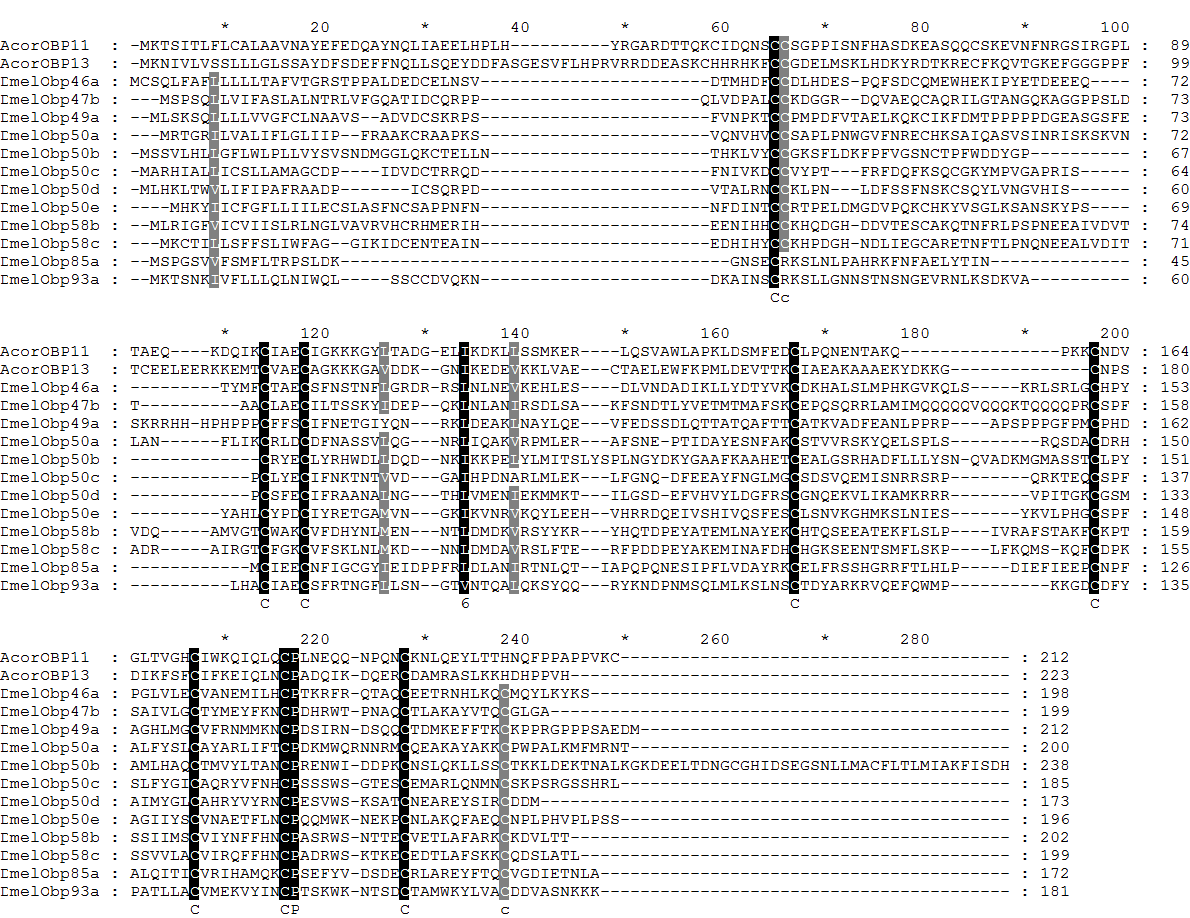

Supplement: S2 Fig — The alignment was conducted using the ClustalW 2.0 program. (TIF) [file pone.0121504.s002.tif]

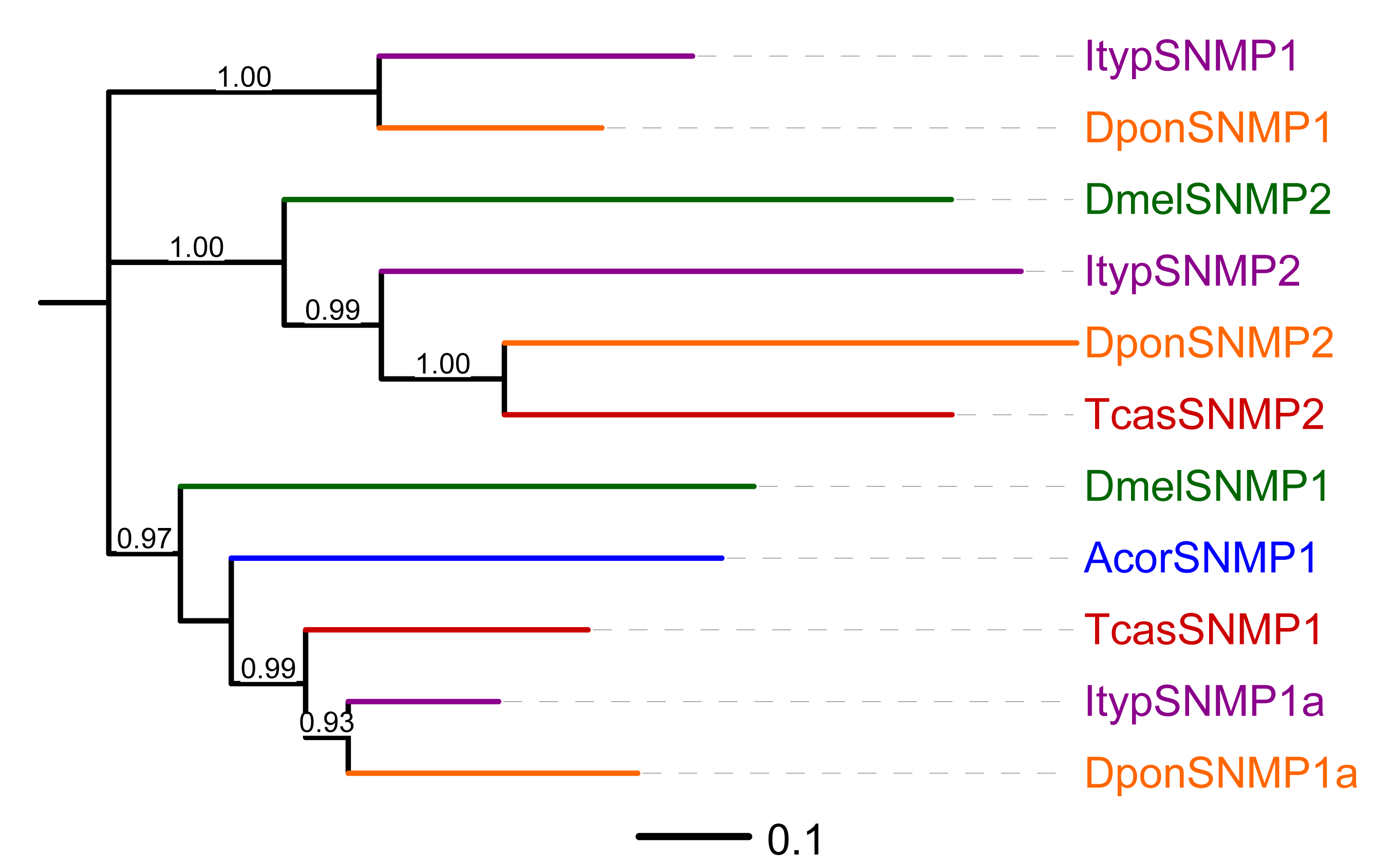

Supplement: S3 Fig — The tree was constructed using the FastTree 2.1.7 program. The numbers above the nodes indicate support values. Support values < 0.9 are not shown. (TIF) [file pone.0121504.s003.tif]

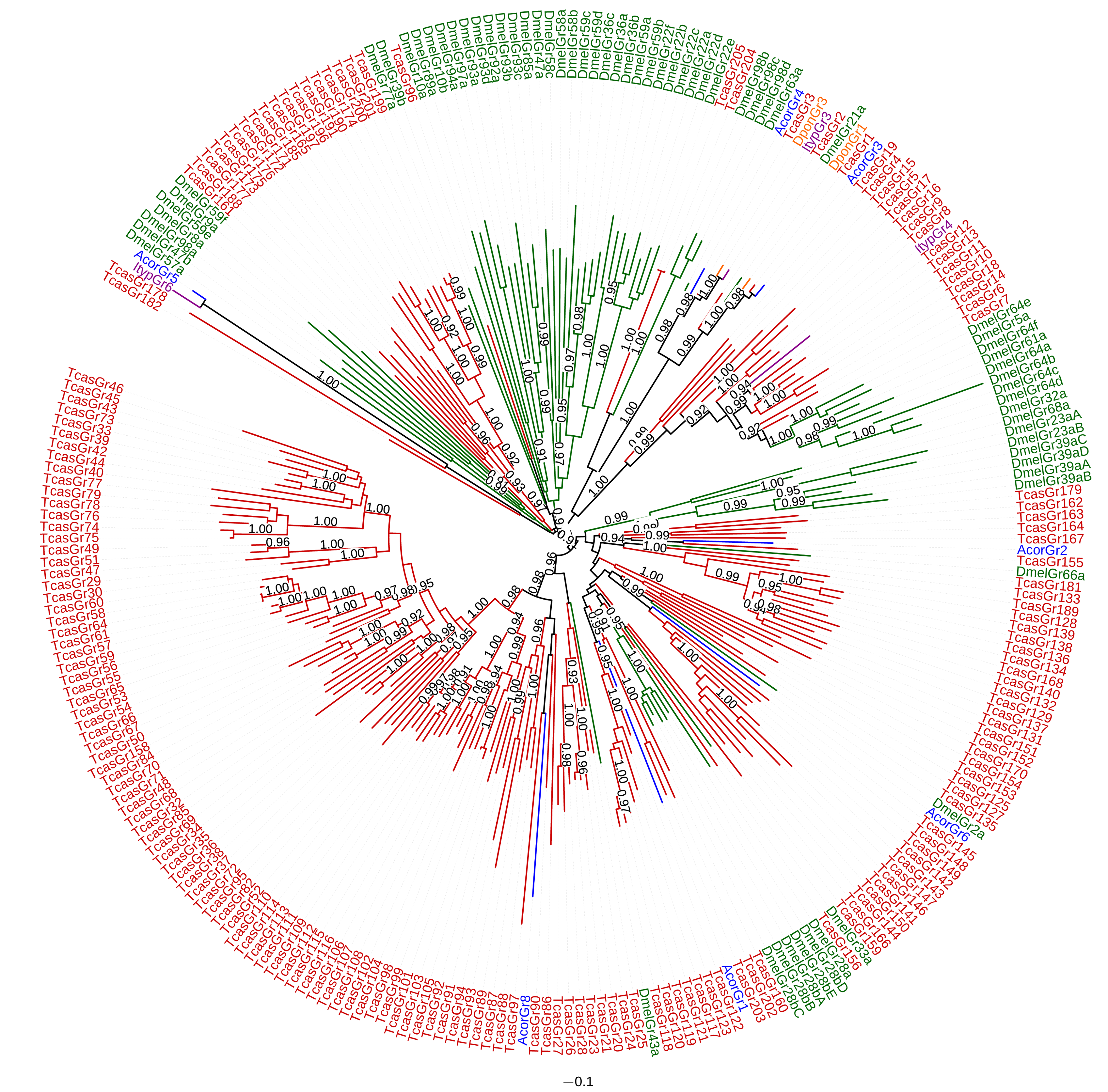

Supplement: S4 Fig — The tree was constructed using the FastTree 2.1.7 program. The numbers above the nodes indicate support values. Support values < 0.9 are not shown. (TIF) [file pone.0121504.s004.tif]
