# Supplementary material for: Chemosensory Gene Families in Adult Antennae of Anomala corpulenta Motschulsky (Coleoptera: Scarabaeidae: Rutelinae)
Source: PLoS One. 2015 Apr 9;10(4):e0121504. doi: 10.1371/journal.pone.0121504 (PMC4391716; doi:10.1371/journal.pone.0121504)
Supplement: S1 File — (PDF) [file pone.0121504.s005.pdf]

S1 File. FASTA format of the amino acid sequences of the *A. Corpulenta* chemosensory genes.

>AcorOBP1
MLKLVVLLTLGIYVPTVMCMSEEMEELAKQLHNDCVAQTGVDEAHITTVKDQKGFPDDE
KFKCYLKCLMTEMAIVGDDGVVDVEAAVGVLPDEYKAKAEPVIRKCGVKPGANPCDNV
YQTHKCYYDTDPQSYMIV

>AcorOBP2
VIVSVQALTGEQKNRIKKYSLQCLTETNADLTLVHKGQKGEFVDDPKVKAFVFCLLKKSQ
IVDDDGYPRPDVIKEKLSKDIPPDVITKVLAKCNPT

>AcorOBP3
MKYQIVLLFLASVCFENAISLNEAQMKAGIKLIKNTCRTKTKITDEQIAKMHEGVWDDAD
DVTKCYCHCALGMMKMQAKNGAFEYELFEKQKPMIPETIRETLIASVDNCINAGEGLTK
KCDLSYAFFKCVYLYDPEHYMFP

>AcorOBP4
MACISWRYFILLLFSVELTYCYLTEAQIKATQKLIRRTCKTKAKITNEEELDRLPKGNWDD
VSHTSRCYLHCCLSMLKVINSDGFIDLEAGMRQSAILPPERRASSEIAIDTCKDKGEGLTDK
CDIAYEIAKCLYDFEPKFYLIP

>AcorOBP5
MMKLLLLLVFVSLSMQFQAKRKLTQDEIRAANKKCLKNSGMDSGVVKNIISLDTFPKPSD
KYFKYLECMYFDQGYLDSDGLISYETIEDFILDFYDVDTVKQALEPCVVLQEGQNGGERA
YNAAKCLIQNLEALEKRYEKQNKNADNTT

>AcorOBP6
MRSIFVVTLLFGSFLSIHADAEYEAEKQKLRQKAVGLMTECKDKVGASAEDVQALTNKQ
LPTTDKGFCLIECIFTNGNVMKNGKLDVQGTLQVLDTALSKNPDAKKKTTAVLQTCEKEV
GAGGANGCETAKLIAECFKKEAKK

>AcorOBP7
MYRLIILFLCVLGANATTKLLAIFQEHALKTGLDCLSEVDATMDDLKSIINHDMPTTRAK
MCLITCIHEKFGIQDANGKMLKDQTIAFLDVLKDDPPYHNLAKDHFLHCLETVSETDEKC
TIGANLMRCIVVGGNEKGIF

>AcorOBP8
MFCKLFILSLCLLNVVCVTKLQRMIKDAIAHLGTECLSETSDATMSDVQDLVDHVRPTTR
KALCLITCIHTKAGMQDEHGKLKEEGGLNFVEPLKQEDMDYYEISKEHFINCINTVPDDA
EACIVGGRFNDCIIIGGKTKGILD

>AcorOBP9
MKQIIVLAVLCVIVSVQALTDEQKAKLKVVSDKCIASSGADPSSVEKGRKGEFGDDPKLK
EFIFCLLKATEMLDDNADVRLDKIKAKISKDLTEAEIDTLLGKCKPTATVPVEKAAEFWK

CYWANTPKRIELV

>AcorOBP10
MKTIAVVVLLFASSVLCQDSAEDRQQRIRRYREECVKEAKVDPALIDKADAGEFADTKEL
KCFAKCFYVKAGFITEQGELLMDVVKAKLPPEHEREKALAIIELCKDLKGADACETAYAI
HKCYFQNAHAANLHKN

>AcorOBP11
MKTSITLFLCALAAVNAYEFEDQAYNQLIAEELHPLHYRGARDTTQKCIDQNSCCSGPPIS
NFHASDKEASQQCSKEVNFNRGSIRGPLTAEQKDQIKCIAECIGKKKGYLTADGELIKDKL
LSSMKERLQSVAWLAPKLDSMFEDCLPQNENTAKQPKKCNDVGLTVGHCIWKQIQLQCP
LNEQQNPQNCKNLQEYLTTHNQFPPAPPVKC

>AcorOBP12
MCSSVAKFYILGIWIYSTLILTKALECGLSSSQNQDELRRYTDICMKKNLPINGDQIIENTSM
GQQGYDSSYEDDSKPSSSEDSMSSKEHAMNSLREDSINDSMNRNGDNNLRNNTEITDDVI
RCVLKQLGMVDPSGYPDHSKISQNLIKGAENRELKDFLQDSTDDCFQMMEQDEHMDSCS
FSTQLIKCLAEKGKSNCADWPMSDVPFSHLF

>AcorOBP13
MKNIVLVSSLLLGLSSAYDFSDEFFNQLLSQEYDDFASGESVFLHPRVRRDDEASKCHHRH
KFCCGDELMSKLHDKYRDTKRECFKQVTGKEFGGGPPFTCEELEERKKEMTCVAECAGK
KKGAVDDKGNIKEDEVKKLVAECTAELEWFKPMLDEVTTKCIAEAKAAAEKYDKKGCN
PSDIKFSFCIFKEIQLNCPADQIKDQERCDAMRASLKKHDHPPVH

>AcorOBP14
MQKLLVFVVQMCLILSIQALTIEEKATVTKIGKKCIEETKVDVKLVEKGERGEFADDPKLK
EFVFCFLKASDIINADGYPKPDEIKVRLANDAPVSEIDDVLSQCESKAATPVDRAADLWKC
YWKKSPVHIPLQ

>AcorOBP15
MKSLVVVFVLLGLYSPVILGETLKEHGQKVMEKIIDYATSCADSLGVSPEDIKLLMEKKLP
ESKEGQCIPSCVNKKFGLQKADGTIDKEYRNSDMEEVKAIDEEVYNKMNSIWDKCVLNG
AEGSDECDTGIKLVSCMKEESEKVGLNKEAMGF

>AcorCSP1
MEVTFLFSTLIVGLFYVSEAQDNSQGLFLWKYKVDVNTVISSKRLLINYINCLLDKGPCT
TEANELKKILPNAISTQCKDCSITEKQAVGKIFAHLLQYHRDLWNELLDKYDPDGTFRKQ
YELDEDEDYDDEKESN

>AcorCSP2
MQLVQIFFVTCIAATTLAMPQNRPQVSEEAIDRALKDTRYLMRQLKCAVGEAPCDQVGRR
LKSLAPLVLRGACPQCSPGEVKQIQKVLGYVQKNYPREWNKILQQYAG

>AcorCSP3
MIMLLYLLLSVILIVPISGGDEKYTTKYDNMDIEHILSNDRLLSKYVQCLLDLAPCTVDG
LELKKNMPDALETNCSKCSDTQKVSSEKIISYLIDNRPDYWTPLQKKYDPTEEYTKKFIE
ARKVKAKVST

>AcorCSP4
MRLLTVLPLIVSSSVVLLVMGADPPGGYYTTRYDHLDIENILNQKRLVHYYAACLLEKGP
CTPQGTEFKNILPEAIKTNCLRCTEKQRIVTTRTIKRLTKEYPDIWGQLEQKWDPTGANVK
RLLASVNRPRPISGIPSLADRFGNEDQNNLGEITTRSTTSSSTIGGGISSTTSSSVSVPSSTGSS
SSSSATTTTTTTTTTTTTTTRPPTTTFRTIYKPVTARPFNSIGPNLMILNPKVIIDKVLYTADA
VLNTVSGVLKG

>AcorCSP5
MEKCFSFIFVLCLVVVAIQAQNRYTTRYDSIDVDSILSNRRILTNYLKCLMDEGPCTNEGRE
LKKTLPDALANGCSKCNEKQKSSAEKVIRHLIKNRSNDWKRLTAKYDPSGQYRKKYEAQ
YNIKA

>AcorSNMP1
MQFHARLALASGIILTCITIFGWVAFPNLIKSKIKTGVTLKPGSQLREMYMKIPFPIPFYIYVF
NITNPNEIARGQTPIVQEVGPYEFEEWRSKDFVSENEDDDTMTFYSKETFIFKPGTLNGNE
VLTIPHPLIVAILLTVMRDKPSASNLINKAMNSIFRNPAHPFLTATFREIFFDGVIVNCSVTDF
AGKAVCTQLKTEGAKDFQFIEENVFKFSLLGTKNGTASNKFKVKRGLKDYRQTGMVVE
WNDLTELTTWTTKECNQIKGTEGTVFPGLIPHGSDIPSFAAMLCRSMAAKYLGDSKYDGIP
VSRYTLDFGDQSSNEEDKCYCPAPDECMKKGALDLRKCSGPVVATMPHFYNSDPSYVNG
VKGVNPDKAKHELYLLFNTFTGSPVAARNRLQFSMPFVPMPKVDVMKNVPNTLIPMFWV
EEALDLNKTLTKPLRTMYTTMKVVRIISWIILLGTIGGLGYAGFLYYKRSREIQITPVKKDK
KDNAISTVMSKSADDKGGVSNPAVSDNEIDKY

>AcorOrco
MMQFKPQGLVADLIPNIKLMQFSGHFMLNYYAETTGAVHTLRLGFCFGHLFLLLLQFGFT
FGNLVQQSDDVNDLAANTITVLFFTHCITKFVYFAVRQKLFYRTLGIWNQSNSHPLFLESN
NRYHQLALTKMRRLLIVIMIGTIGSWIAWTTITFFGDSVHTRKDPNNENETITEEVPRLLVR
SWYPWDAMSGAAYYVSLVYQIYYVGFSMLHSNLLDSLFCSWLIFACEQLQHLKEIMKPL
MELSATLDTYVPKSADLFRAPSASSQDNLVDSDYNQSNEDANLRNLYTTHQEMGVTYRS
GNLQEFSSGGIGPNGLSKKQELMVRSAIKYWVERHKHVVRLVTAIGDAYGIALLLHMLTS
TIMLTLLAYQATKIDGVNKYALTVIGYLLYALAQVFHFCIFGNRLIEESSSVMEAAYSCHW
YDGSEEAKTFVQIVCQQCQKAMSISGAKFFTISLDLFASVLGATVTYFMVLVQLK

>AcorOR1
MSWGYKIGSFLNSKILKNEDEVFQDDAKYITMFSQVFAKIINLWPGDDGVSKKFTFGLML
TSVITQELSLVMYLLTTKINVDVVITSMASMIILLQSIVKSCVYFYNARKLKKLIQTVRKEF
WPANIMGETTHNDIKYNSKILSLVFVVQYASAILFLWFSVLLPLAKPGRKLPHTSWFPFDST
VSPLYEIIYVWEVYLTAYINANIVCSYDTLFCSICGNCISQFRLLSAAVKCIGISGKENQISNR
LLRLQGVDYNPVRTNQGKEDRESRRLLVICVNHHQKLIKITQELNEVFGPGHLAQFFASAL

GTCTACYKIPIEKNPGDLFVLIAFYIAHVSQLLEYCALSHELSYWGIKLGDAIFESFWYIKK
HSQIRKCLPIIILRCQRAISMNALGIFELNYPSFLIIMRFTFSLYTFFNNMSKSTI

>AcorOR2
MVSKSYFKVQIFCFKMMGILMEDIDWNRFTSRAYAIYSFLLLSCFIYLFIITEAIDLILKWGD
LDNMTFNLCYLVTHFAGLCKIAVIMHQKSKIRTFYQSLESGYFLPNHERGGNEEFRIISSAI
WQSNMQTYVFYTFVTVIVANRGFYAGFDKGYFIQFTSVNGSETTNKHYKVMPYTTWIPF
DTNVSPYYEIAFAYQIVSALIYGLLIGTCDSFIAGFMVHIKAQLLILKNSFGNYIYAAKVKT
QVKNDFENLSFLKNCSDNGLKTNKDIKNLNPETLIYVQRYLRDCIIHHQQTIKLVEIVESEF
NYLMLIQFLGSLLLLCLSLFQLSINDIRSTRFFSMICFAGLMLFQLLIFCWNGNEVLVESLEI
AFAAYGSDWFLCDLATQKALVLVIQKAQRALQLSAGKFAYLTLETYMNILRASGSYYMVL
RKVNE

>AcorOR3
MDDENSNGNPDVGADFFKPFFSILKVILYWPLYTSKSQLLQHVHMLLSAVKVTFTVVLYIF
LECFTVYKRSNLQERLAGSFMMFTDVSYLTKVLFFIINRKKILLVFKAVSDDVFSPKDTLR
KEMITTTMTNWKKVFYIYITNCCFTVTLWAIIPSLQNGSIVLPYNYHYPFDVTSSPAREFAYI
YEAGILYLIVVSHVSLDALMVGIMAFISAQLDVLNYNLRGLNNLDEVNSYSKHQFLKEEQ
IRCMIFHKKVMRLVKMLNQILAVPLVTQSCFGAIILCLSLYKLSTLNPLSGEGLSFINYYFG
MLIQLSVYFWYGNEIIWKSNELSRSAYQCKWVNTSKPFRRHLLFFMLSTREPLKIYGGRVL
ELSLQPLISILKFSYSCYTLLKSV

>AcorOR4
MEKETLQYNAFDLERRILWFYGIYFSKDFRPQKLHYLRVIATSFVINTLVLGIVMEMIVDH
NNLETVFQSIYYIIVVIIGQIKTFSLYRSLSQFNSLEDMLQDVIFNAEISTGCTSISKAVDTYKI
FKRIFWGTASFCASVYSLMPLLSGDLSIPIWYPSESFKLYCQLFEIVCIWTIAASLLSIDVIIM
GLIYFMSAQINTLNYNLRNATDRNPDYDAGTQEKQVQDNLRICIRHHLAISEFVSKLEEIF
KFGLLLQIFSSIIAISAGGVYMVFVTLTPSSFIILVSSMTMILAQIAMYCWVGQGLLTESDQI
GESCYMSEWYTCNIATRKMFFIIMERSKRVISFKAGNFFELSFATLVMIIKNAYSYFTVIITAF
K

>AcorOR5
MVEMRFFDLNVRILKLSGLWVPNFTNKWKYRRTIAYNSICILYSMIYFTIAELISFKESAAN
LNDLVKNLNMLMSFLLTLIKVIVWFRYRKDILKIIRFLETPRNVFKDYNLNNEEIILKELEF
KDTWTKSFFIMSTLVPLSAGILSITETLTTGEKYVVFRNDSSLIYIQKLPYYSWIPFDHTSSK
CAFRIAVVSQCIALLNCGYITVGLDMLFVALASSITAHFMLTKEAFRSIGNFGSNEFDNAQN
DNYIKFKNCMMHLQTLIKICQRLEVIYSFLILMQVLVSLVVLCTCLYLVSSIPVGVRLLGNE
LAYLLAIEIQVAVYCFVGNKLTDAALQIPLAIYECNWLNTTSNFKKAVIITTIRMQKPIYITIG
KFSPLTLNTFVMIGKTSYSIFTVLKSRN

>AcorOR6
MKRDITQYNALNFERKILWFYGFYSGKDYLARKHRNIPLIISSCFSFTFITGMVLKVLEYQ
DDLETIFETAHACITSITGAIKLFCLYRALPHFNILEESLIDPIFNLEIFEGSNFISKAVKEYVSF
SKFYWTMVFITFVLYGVFPVISGEIPIPIWYPADCPKFYVQIFEVISILILSCSYPGIELVLFGL

FYLMSAQLDTLNYNLTNSTQWNGEDNREVQEEKIQDRLKCCIEHHLAIIRFINGLKDIFSF
GIFAQIVLNVLIICTSALQFLRNSVTIVTLLSSIMYTLTILTQIGMLCWVGQNITTKSSLIGESC
YMSDWYTYSVSTRKMFFIIMEKSKLVISFKVGNLFEISFKTFIMIIRSAYSFFAIVITMYK

>AcorOR7
MSLRDEKDPEILSDYFWFHKLVLKICGVSFKEQENLIYKIYSRFVQCLIGFLIFGEIYTIILSR
NDLQLMAEIISVSTSHFLGLFKLSVLWKNRASIADALNALHTGVFLPNSRRSGLSEKILIKN
CITKVYMLIAFHSATLVATVFNIIGSSLVTKFKFDDYELWKMPWIPFRLFPITTTIVYYTIYV
YQTVTLILFACIIITTDLMVVAVLVHITTQFHILASVMRTLVENNSVNYISEQYDLYLRKKLK
YAAYYHQELIKLTDRFEELFNMLVLAIFMGNCIVLCFGMYLMSSGELELNQLISEFTYLITV
VMQIFLYCYYGNMITEASDAISFACYETDFVGTDLRFQKGLLLIMMRSQRPIVLTAGKFAQ
ISLAAFVAILRASYSYFMVIKSSSVEA

>AcorOR8
MEEEYVMFSGLYSLNLVGLHPFKSSFKKGVVALIFIVFTLICYALNLAGVILKYDGLKSLA
DSIDAVPAGQQVMVKLLSVLFLRNEMKHLYNTVEKKWDNKIYGEELENTIKKLSLKFKKI
YNTYRMTIWVTAILYVSKPLILFSRTLLTEMYIPCNLSQNYCYISFIAIQTIYIFDLAFVVYTF
DGIFYAFLFYVYCELEKIKYGFANLNVSITTDLNNNEEDCYTKFCEIVKHHYSMIKFLQDV
NKVYYLQLLNHFVTITATIVFGIFFMNMDGFPPALGKVSRYIPYLMSYHFQLYIYCMWGQ
QVFDQVCSVCDVIYQSQWYVRYQPKLAKGMLLVMKVSQIQNKLTIGDMWKLNLGTFMS
VIKTSMSFHAFMQTVYKSDEVLVESYNQTIF

>AcorOR9
MTDRPKLLDFTKLPRRVLWCFGFYFGDDMEEHIFHRLICIISLTLTIPFPVMIMGKILLDLRH
DLQALLETLHYFFLHFWIVVKIMLFLYGFRRLRNLEAWLQTEIFNSYTRRQDHFLNKAMR
KQMSFFKLLWSSTLCFTSMFALFLPKDKVSLNIPIWMPFKMDRIFWHIYEVLCYVITASTYP
AIDCIITGLVANMTAQLQILGDNLERIHLDHEGGLLKCEDKIQERFKRFIQHHIAILQFINETE
AVFSYSLFCQILFSVLGICLSGFQFLLVPPGNTKFMLACGYLTVMFFQIYFTCWFVEDLIIQG
SDVVTSCYASEWYNYGSTTKKLLFILMERAKKPISFRAGYFFTLSLATFVMILRNSYSYFAI
LRHVYKE

>AcorOR10
MDSYIPNFFKVNVTFLKHGAVWSPKDKTQRSYKVYKMYQVIVLFLTLGCSSYSVVMGIII
HLKNFAILIEVLSVSFTILLAAVKTSFWLIKGEKIKIIMNRLETDLFHCEKIDDFDPEGMLNQ
AKLSGIKYACLLLFFSHLVLGLGYIPVMSLACWYYFKDLQITDVPTFKTLPYYTHIPFDHD
TPLKYIFACLLQCVPMYLYVNAFVGVDSLFMNLMNFIATRMLILQGAFRTMRKRCLQKIIG
YDLAPDSLHNSNEMEEYMMSDMKKCIQHLQLLLRSCKDIEDNFQYVSLVQALGTIYILCS
TLLLLSTSPPFTKEFGRNIFYLLGVIVQLGLYCWFGNQLTLKAANVPIAVWESQWLETRKP
FKICMLLTMMRMKRPLLINAGKFVPLILDTQIAVLKGSYSYYTVLKGMSK

>AcorOR11
MAFYKISIKTSLTMLNLKGLNPLISTRSSNTRAITFLLTEVLATITVASSLFTKTLEADSVVD
NISGIVFSIQTICKEVTMLLCRDEFVALLNYVEEFWPVNEFGIESGTNIRNIQKSTSKALKIF
RCLLLMCTVIIISEPFFAEGRQFPVSWIDISCIQTSLICYGVIYGFLCGCTIGLVIFLSLIDGLFF

NLLSYGYCELEQVKYALFNLSIDGDVRGDQVETLREIAVLVRHHVTSLEYVQRVKKLMSK
VMLYQFSSSLFTLCTGLYVLTYQGFPPSVEAAVKFVPFVACAAFQIFAYCVAGQKISEQTESI
ANAAYECRWWFKHQPRLQRSICLIIQRSHRRIVLSAGGLWNLDMDTFIRILKASFSLLTFM
QTMYIPE

>AcorOR12
MNSTSIYKNYFKVHFFVLTCLGIELKPMKGSIANFIYKIYAVMIFTVVYLYFPYSEILSLVYE
ENFESAIYNLAFLLTQILGLIKISLILIYKKKIRMFCKFIETPPFLPDQNRSGEAEFLYVKEAIR
ACNTQGYIFYGLTAGIISQMMYDALSNPGYTKCFVDSATNITITKHIRALPFNSKLPFETIDS
PYYEIACIYGSLSGAIFGYSVGAMDAIICGIMCHIRAQLLILQECLKTFIPRGIYQMRENVKL
TNNDQKLLQSITNNLNETIEIPNTLQKYVHIAVCNIITHHQKIIKLAQDAEELFSPLMLVQFL
FSLGILCFQLFQLSITDIESVHFFGMSSYLILMLFQIYLFCYRGNEIMLHSHNITDAVFESLWF
LTDLKTQKLLLIMMIRACRPIKMTAGKFVFLSLEAFVSIVRGSGSYFMVLKNTNAPATEL

>AcorOR13
MASNKTRTLCKNYFRIHYFVLMLLGVSIQPMEKNIFSYLYKFYSIVMFTIVYIYFPLAEILYL
VYNTDLENITSGTTYICTHTLGTLKIILILIFRKRITAFCELIETKPFLPDPHRSGDIEFDYVQE
AINACNYQGYTFHIFVVAIVLPKIFYSLRDSEYETVFNDFQNVTFLVQRQRAGPFNCVMPF
NTINSPYFEITAIYQASCAAILGCVIGSIDAIICGIMCHVKAQILILKKSLGTYIQQGLFLMEE
DNIDGKNVIGVDEFEMIRNSKTSIQLENVPISLQKYVDISVTQIIIHHQKVIKLSQDAEETFS
LLMLVQFLFSLSIICCQLFQLSILKMGSAQFYSMCFYAMLMLFQIFLFCYRGNEVIVHSYDL
IDAIFQSNWTELNLKTQKSLLLMMTRACRPIRMTAGKFVFLSLEAFMSIVRGSGSYFMVL
RSINMPEE

>AcorOR14
MDNFFDVNFTMLRMSGIWIPDTSSQPIIKLLYLLYNTLWICYSCLFFCPSELVYFANTVTYV
PDLVKNVNMGMTHFLANIKVCLWFYHRKEIMAIIETLGIYGRRYESYGDFDTDKIVQNAK
RFKDIFSVLFLNFAMFTSISSCLICFLNVITAEIPPGEEIDMKLPYFSYVPFNYKASKVAFSIAI
WYQFFPVFNYAYIIVGFDTLYTAILGYVSAQLDIIQGAFETIRPRCMVRLGLKLSQNILRDPP
TLMDEMHKEMNKVVNHLQVLLDICRRLEEIFTNVILAQVMISLIVFCTCIFLVSNLPMMSL
NFAAEMIYMIAIECQLLIYCVFGNKVTVSSGNISSSIYNGDWYSTSTSFKRSMLITMSRMQ
KPIYFTIGKFTPLTLSTFLTISRASYSFFAVLKNSDFSN

>AcorOR15
MDYNYKNIFNLNLTILRVLGYFPSNTGSKPFNAMYKSCTCIAYFLALLFIASQAVEMVLMA
KAQNLEKLSTTCLKLFLNVSYFVKLTFFINNNNRVKLLIRKIEHKLVSPSSPQQDESMTKHI
KCMTTFSRTFLYMSVITCVLFAIFPLIDKNEELEEINTDGWYPFKSSNTVTLVAYVYLSLEEL
LAGLCNVSMDCIVIGCLSYICMQVRFLKHNLKHMKDICTNTLAKIPDQSLLNKNDYRRQL
QEHMDDTLINCILQYQTIVRIKRDIEEIFGMGIFIMFMFDCLALCMTMFQLLIISFKSIQFFC
VIIYMMCITMELMAYCWFGNELLVISSQVPVAAYESDWIDTPVYFQKNLLMFITIAMKPM
KVTVIHFSLSVETFTVIMRTAWSYFAVLRQKYNEEH

>AcorOR16
MTRREMNYPKNYFHKPLRNLALCGLWLYEPKKMNYKILHYLWFIILATCACFYLLTEYTH

IIKHLHEMQEVTLALCYIFCHSMIFGKIVIFIIKKGKISKMVKLLESGPFLPNVARGGPEEIDII
RRTIHLTNVQLKIFGAVIVVMMTTGVLPYLKNGRTFNEISPNITQVIVKFPYPSTLPFEVDYT
ASPCYELMFTFQVFSMNLYGWYFSNIDALIIGLMMHIIAQFKILVSAIENVTKRAENMAAH
DKSSALSQVIKKITFIKYNIDCDNFIIIERVETYSETIMANLKKCINEYAYYHQEVINLVDDM
EKSLNFLFLIQFIGCLLTIVVGLYQISLVPFGSSSFTNMASFSFAITFEVFMHCIYGDEISFYSA
EVGKAAYNCEWIKADDRIRKNLLLMTLRCQRKCFLTFGKFSKINLVLFLSIMRGAFSYFTF
LQKMNEELNM

>AcorOR17
MSEVSFKSQKVAQYDTIDVSRKILWAFGVYTGKKYPNRILCKISLAINCILTFTFMISMLINI
LLNMDDLETVFVITHLLVTELGYTTKTYYFMRMLKEFNALEELLEEPIFNDHSLEQDNFV
AREIRTSKILSNIFRCLSWCAQLTYTICPILDGDIWAIPIWVPLTDGSPKLIYQAYEALCFMSL
ASVEPALDLIPVGFISTMAAQLDILNDNLKHSADKNEDELEEGKIRKRLAKCVKHHLAILS
FLKKLEEIFSFGIFIQIFTSVGAICMSGLQFLVVPVRSATFVAVFIYFWVMVVQIGTCCWVGQ
TLITKSNQIRDACYESTWYNCNTSTKRIFFIIMERSKKTISFRAGNFFNISLATFVMIIRNSYS
YFAVLMQMYK

>AcorOR18
MDLLEKYSLRIMQLRSLNARESSIVNRAKAIGWVFVDLTFLGSTLYYLLFHVTDIIEAVDCI
TVIIVVCQTLMKQLSLLIYQQEYADILNTVDKFWAYDKFGAAPNKKLTSIQNLIEKLVQCH
MVVIIACGFFYYFKAALQREKVLIMGWVTVCGIENNMCYAVNYAGQVMWIAWLMPIFLG
YDTMSLLLLGRVYCELEQIKYGFINLEVQGEESEVLKQVSALVRQHNLVLDFLEKIGGLFS
SILLCLFLTVLMALCTSFFLLTATGFPPSFSVLSRLGPYLAGSCGQNLLYCIVGQIISDQTLSV
ADAAYDSKWFATKSLGLRKAICLVIQRSQRSTQLAAGGIFNLNLETFVAVTKASASALAFL
NTMYN

>AcorOR19
MRSEFSTYTNFLKVLLYWPIESKNVYIVIAYDIMSSIKFILVYIVFFIMDCIVIYENSANVEQ
MVSESFLVFTLCNFFVKSFQYTNCNRRIRGVLEDISLDIFEPKDKQSEDVTKKAMAYSKRIF
YIYVITCCSFGFLLGAGAIISEEHNALPFNFSYPFDKTHGFGYKVALAYEALSISSNAMTHA
TMDCVVYSILAFVRVQLSLLNEDLCNLGSIQYNTYQASLEQQILCIEKHQAIKRIVKELNEI
ITFPMFVQSTLSAITLCMSVYKLTGVELLSEEGISYIMYCAVMHAQLGIPFWYGNEIISKSN
ELTISAYQCNWIEENKHFKSNLLIFLMSTIKPIEVSGGYFIVMSLEPLITILKCSWSYTTILRSI

>AcorOR20
MLWVFGLHLLNFKETGFRLILFKIRTLITIVSSTTMLLFLLVKIIQSKNDLMSAFETCYYSLI
QAAFVIKLYIYLHYLPILIELENKLESNIFNGHKQDQLHFISDAIKSHQTYLGFYKICCVSTAI
FYSIFPALDGQQLAVPIYSPLDLKKYRLIVYLYEACNFFITACNNTAFDGTVIALITIMAAQI
DVLKDNLIRATLRDQTVDAKDQERCIHKRLKHCVIHHDAILDFTKTTQKIFSNGVFVQILV
SVLGICMTGIVFLTVPLKSMKFISMVLFLITQVVQIGMFCWFGENIRAKSSEIAQSCYMAH
EWYSNNMSNKKILFIIMERAKVPIIFKANGVFVLTLNTFVMVLRSGYSYFTVLRHISQNMD
LS

>AcorOR21

MDTLEKYALRVLEARGLNPVKSSIMSKVNAIFWTIVDGTFVITILMELVSNTSDIFTFVDNF
SAITVASQVVTKEIGLLTHQQEFRTVITCLKEFWPKDKFGKQVKAKLDNIESFSKRFLQIYI
CSVACAVSLYMLKPILEGNKILPIMWVTFCSLEESLYCYIFNYILQVVWAACGLHMLVGFD
CLFILLLLCGYCELEQIKHALISLDPDETTDEDDAPLLDLIASLIEQHNRVLNLLKRIQDLLG
SLLLLQFVATLLSLCASLFVLTSVDFPPSLPVVSKSLPYIFSVFTQNLIYCVAGQVISDQTLSV
ADAAYASKWWIKAQPQLRRMILLMILRSQRPEEMTAGGVFALNLETFVAIMKTTGSALAF
MNTVYGEEE

>AcorOR22
MPVPKPLIRKLSAEFSKSMYKDDVKRCILPGKILLQGVCCWPDDETLFYKSLGWFLFWNL
IIVEIFHAAYVVKNYKDIEDAVTAGATVTTTMEGIVRLHTILTKRNIINSILVKVWKRFWPL
DVVDPIKRIQLRKRAQLALVLTSIFLASSIISNSQMVAVPYIKNRTMLLKSTFPFDWDQLYY
YEIVYVWHYFSDWFVLFMINSFDFFFVALVTICSIQFAIMQEVFKLILSKQSLRHRAVIFGQR
GKTMDDKEMLLKCLEQHQLLIGICNDLEKSFNITILIQFFVSTSAICAASLLLKVDYSQFLK
MLMYAAAHLSQLFYFCFVGHELSYESGQLSDAIYECNWHLSYDRDFRKALILIIQRSHRV
QWLTAAGMVKLDFATFLKIMRLSFSFYTLLHDMLMKNLDLN

>AcorOR23
MEKETLQYNAFHLERRILWFCGIYSGKDFQSQKRHFLRAMGVFLVLLIFDLSMLMKIIVD
HNKLEIVFQTAYHIIVIIIDQIKTVSLYRALSRFNNLEDMLRDPIFNTETSTRCTFIAKAVNNLI
NLKRVVWRMTIFIATLYSLMALINGNLPIPLWCPSIVLDMFNPYYQLYEVLCIWVTTATIST
DLILIGLLYLMSAQIKTLNFNLRNVTDTNPDYDAERQEKQVQDNLRISIRHHLAISDFVSKL
EEIFSFGLFLQIFSSIIAISAGGVYMVFVTLTPSSFIILVSSMTMILAQIAMYCWVGQGLLTES
DQIGESCYMSEWYTCNIATRKMFFIIMERSKRVISFKAGKFFELSFTTLVMIIKNAYSYFTVI
ITAFK

>AcorOR24
MLEVLYTLGEYTGVFPQKNKTDDYKNVLNILVVFYAAIGVFWFGVNVLMNSKATLLDM
VDAIYTMESECSLLYYYMISIHTRKPALKLYNDVTDFTTFGKPKTLEREETRICKIYKIIVGY
GIVAPTVTNGFYLATYDWCMRSHRNDDDIQYCGYTFGIYYPYNFEKGVSFIIHTAINWYSF
VFLSLVALTLVGYTICVARYLVLKIDHLNTMLSEVLKNDAVNRRELLKKCIRYHKHIISLVD
GLNELHSMNNAPAIFLYSTIIGVCLFYLTNEYNTKAIALACGYIGGIFCLNFAGQMILEKSET
VGVAAYNMEWYNADSSTAKDIMFIIIRSQVPLKYKAGPFGTMSLIFFGSILRGAYTYMTM
QTDIKEK

>AcorOR25
MEEELPHKIRRLSKDFNRDISNDGAKRIMIPGKIFLECLLVWPDREMKYITVFNWFMFINVI
IFEITHACFVVPNIADYTTVIAVLVTVTATFQFLVKFYVIVFKKSIINQILLNIWREYWPLSVL
SPKKVKRHSSTCKVKLRLILGCYTLAVIFAAIITFAPFLTNTELIVKSIFPFQWNKTYTYELV
YTWQFVTAWYITFLINSFDMLMISVVIISAVQFAVLQNVVKNILTEKGERQRRYLYNKDISN
QDMFKRWLEQQRMLIDTCNKLEEAFRIPILHQLFCSITGLCASSVILKVDQSKFLEMSTIAL
ANMFQLFYYCFASNELTLQSEKTSDAVYFCNWQISQDMKFKKALVLVLQRCQKPLSLTAA
GFIDLNFLSYIAVLRLCFSFYTLLTDLIVSKLEAAELQ

>AcorOR26
MASQQELSFLNRFKIFQINREIIMDGAKYLLIPGLVSAKIIMAWPEDVRSKSFEILVFVMSFI
QCVTIITSIVLNIVDVNTTIMMISAFEAVLQVVGKFSALIIKSKDLDKLIKTVRYEFWPSDIT
NKDTAEKIRKDSRILFKIMMIESSICIMVVLTIAFGPLLKTGRVLPYPTWYPFDTSASPVYEI
VYILQSYFGLHLSVPPIIAYDMLYYSLCANCTAQFRLLCDALRCIGNGTEDEMITKLVEFDE
SQKELRGTSEQKLLILCIKHHQRLINTANEISQAFGNGHLVQLMGSASGICTACYILTSNPD
LSSVANALVQYIAHVGQIFIYCAVSNELSYWSTLVPTAAYESLWYKKKYPNIRQCLAILITR
SQIAISMQAFGLFELNYTSFLSIMRFTFSLYTFLSSFA

>AcorOR27
PEDTSFNIYHGLILAACFLNIVSTTISVIVNTEDTDALVLKLFSIGALAGISVKYAALLYKSK
DFTKLITIIRVEFWSSDILDLDCDRSIYKDTKLLLVIIVTEYLAGCTCSSLMAIIPYIKSSKELP
YSLWLPFDWTRSPYYEILYLLQGYIAVFLMNAVFGYDSLYYTMCANCTAQFKLLCCAIKCI
GTGTEHEIIRKLLNIPGLIHEWNPRISDEERILFICIKHHQKLIKMCNDINVVFGNGHLIQLIG
STIGICAACYRITTEPNFNDLLTCIAYYMAYVGQIFIYCAVSNELTYWSSCVSIAAYHSLWY
KKKYANVKQCLSIMMLRSQKPVSMQAFGLFELNYAFFVTVMRSTFSLYTFLTKMATK

>AcorOR28
MTLNQNELQYTIINTERRILWANGVVYTGKQYPRSITNKTVTIINTILTFTFLTSMLIKMILTK
DDLGVVFEIIHMFITEVVWALKACYFVLTTKQFNALEESLKDPVFNEFTRDQKNLVSQQIN
KTKRIGNIFRNMSLTTCACYILCPILDGDVWAIPLWIPFTDGDPTIYYQLYESICFLSLASAH
PSFDMLVIGFLNIMAAQLDILNDNLMNSTDRNEDEDFEVQEAKIKSRLRKCVIHHLAIVRF
LSKLERLFSFGIVMQIILSTIAICVVGLQFLRVSLYSGTFLGIMFYFWTMVIEIGLYCWAGQN
IITKSTHITTACYTSNWYNCSTSTKKMFFIIMERSKHEIIFRGANFFDISLTTFVMILRKSYSY
FAVLVQVYK

>AcorOR29
MSFTVETLQKEDLIHVISFGTRILWVCGLYHCKHLKKHFAYNFARILVIALSLPFPCLIITGL
VVSHSDLSGFLELGMFFLGSSWISIETTAQIYSLRQVAEIEEMLESDYDYFKPKTELQCQFIL
AKRRRLVLITQIVWFCVYSFMIIFVLCPLVTNASLVIPMWIPFGNKEFSAYLYQSFYLLILCG
IYTLLHNTFLGPILMATAQFQILKDNLIHATDRSEDEDGFAQEKRIQERLKRCVKQHNAILK
LVSTVQGMLAPVFLGNISFTILGICFTVLQIILATDSGNFILLTSYLGILILQMFLTCWVGNDL
ISETSDITQACYLSEWYNCTPSTKKMFLIIMSNTQQPISLQSIIFPVSFGTFVMILRSSYSYYT
VFSQVYD

>AcorOR30
MSTKTGKLEKLSLKFTQDIYQDGVKKCLLPVKVLLQSVCCWPDDELPYGKAIGWIFFSFL
FINGVFNATYILMHGKDISEAVGASVTVTINFEALVRIYCILRNRRVFNEILVKIWKQFWPV
KAVDDKTQAHLENKAVFAITVISIVLITSIFSNTFITTMPFLKYNQLISKSTFPFDWNKHFVY
ELIYIWQYFLNWYILFAVLAFDFFFVALVSMCAIQFSIWQHVMRNILNEESKEQRRVIFGKM
ENEMTDKEMLRHCWQQHKLLNNICDDMESAFSITILLQFVVSTCANCAAFLTMKVDSSQ
FSKMLSFSMGHTTQLFYYCYSGQELMYQSEQLSHAIYECNWHLSYDRDFRKALVLMLHK
SQRIQCLTAANFTTLDFTSFIRILRLTFSFYTLLDNLVEDTAENGI
>AcorOR31

IIGIMQTAYVICLIITCSLIAFVAGCTEFIIIRIEHLNLLLKRVVKPNDLDSKESLIKCIKYHIHII
SLVKKFNMCFDKFTMLFLLQTGPTIALTSFSVIVEPKPSVIIHLAGWTMTLFIFCLSGQRLM
DASTSIGDTIYDTQWYKMNNSLGKYVILILIQAQRPLAMRMWLYSEASYMTLAQVMKLA
YSITTLLNSTLQKD

>AcorOR32
RMLPMTMIIVCNFEDNSCFVFYYVLQICGLFTQLITLVGFDGLFFTLLFCGYIELEQIKNAL
VNLDRNGKAGISDEKLLQQTIEIVEHHNFVLEYINKFDRLFQIALLVQFGITIFSLCSVLFM
MTADGFPPSTSNLIRGGPYALSALCQILIYSAVGEKIVEQTEDIAQVAYEVDWYTCYRPK

>AcorOR33
LPPAAILSNTKISLKPLLVHKLLFKSSFPFNSQALYIFEIIYTWQYFVDWFVMFMACGFDFFF
ISLMSVCITQYIILQDVIRAVFSKESKKHRKIIFGERGINMTDKEMLFECLKQHKLLIRICSDL
EEAITTTILLQFAVSVGANCIAFLILNIESSLFVEVFPYCGAHLLQLFYFCYVGQNLTHESGN
LSVAIYESGWHLCYDLQLRKSLVLMIQRSQQEQRITAVGLIELNLESFIKLLRLSFSIYTLLD
SFLVVDDE

>AcorOR34
FLPMEFVKLITSYQNVKSTMEQLGVVTMHMISTLKIVNLYFKRNEISRIIDELHYNDLTETS
GSLERKNLQNKFHRKIRRLCMFFFHMGNCTSTILCATSLIHLIICKHETVYQEFCSTVQPIVI
STPIHIRSLIYSRWIICAFQWMCMFLYGWQIVAHDTLFAAILIKIACNIRILQMDFKNITAESD
QNITKMNYKMNQLTFQLQKLIRTCQCAANVFQYIILLQVLSSLFILITCLYVAASVPVFGIEF
VFQLQYYLTVVTQLSMYCWFADEVTILFSQMPVSIYQNYWICGDQSFKRSMLINMIRMN
KPIYFIIGTVAPLNINVLVYILRASYSYFAIIKNK

>AcorOR35
SDNYCYTFFLVIQIVYVSAMVFTTFVFDVVFHAFLFHAYCELEKIKYGLQHLGISEDVDND
TIVYKKFCNIVKYHNFTLKFLDKINDVYYLQLLNHFATFVAAIVFGIFFMNIDGFPPSPDKL
SKYIPYLITHQFQLYMYCVLGEIVYNQVNSISDVVYHSKWYIKRQSKLTRGMLMVMIVSR
LKNKPTIGNIWKLNLATFMQVLKTSMSFHAFMQTVYKTDN

>AcorOR36
MIKYHSIAAAQINILREKLRTLHTTLDDKFDENVRQGLHDCIRLHISIIRFLENINRVFSEVV
LIQYISAMILVCNFVMQLLFLVDPLSTQFAWSCSFLLVILTELLLYHWFGNEVVVMSHRIGE
SCYLSEWYMFNSANKRMIIILMERAKRPLNVTVYKFTDVSLASFGTVMRWSYSLFAVMR
NMYTKEI

>AcorOR37
DEFNRNISKTLAVRYKIVKFIQLGVVGISIVGVLAFFLRPVFISDVTFMLETWIFIDSNLLAGI
VLMLQYYYFSVIISVLLGYDFIYMSLCIDMISQMELLKHKISQILSDNIANVTLELVTCIRHH
QILLSVYRRMREVYSLMLLFHYFVTLIGTCTTFYEFLGKSDVPDFVINLVTVSVLFLQFGC
YAFPAEQVALEFFDLSNFTYMSKWYECSIRVQKLVLFIMTISHKELCFSGGGIMDINANAFG
SVMRK
>AcorOR38

AKRTHFLYSTVQRMFVTLAILGIILYSFRPLATKGGLVFPSRIFVDLVGFQAVLLFSQYYFLLI
IAAVVPGYDIIYICYSAHVIIQIRMLKYKFEHITKNVEIETINSYIRHHQFMLNIFDRMKGVY
FWMLFFVYSLTLITGCSQLYILILGNTQLSDLLASAVFITALFFEFGLYTFPVEEIVSQFTDISS
SVYKSLWYERALEDRKVLLYVMMKGQRQSYFSAGGLIEINVNTFGSVIRKIFSFYAILKNV
LNK

>AcorOR39
KIASNTKLIYNYTKMVQTFLLCVFITSVHFYFLKPFFNSDDVFPFNVWINFNSLLLNVMVL
ASQYYCLCIVTPVVLTYDVIYFSICLHVIIQLRLLKYKISRSSNNTQNELKIWVCHHQLLSSI
FTRIQEIYSGTLLLQYLMTLGMTCIQLYILNTGQLDVADTTELILYLATMYTEFGYYSIPVEE
MSFEFLDVGNAVYESLWYETDARTKRSMLFVMMYAQDLKYLNGGGLIRVNIDTF

>AcorOR40
SWTTFCDIDHSVCYAFNYIAQLLYVLWGLMGLLCYDIMIMLLLAAGYREFEQIKSGFLELS
IDETVGEENIKALEQIRALVKQHNLVLDFIDGIGSFLSKILMFQFIAIVFTNCSSLFLLSVVGF
PPPASTTCRIIPYLACLFGQNFVYCIAGQLISDQSVSVADAAYGSKWWAKTQPSLRRAICLV
IQRSQRRSQISAGGLINLDLNTFMAVTKTTSSVLAFANAVFQ

>AcorOR41
MPGKKITVRKLSLEFTRDIYKDSVKRCILPGKILLQSVCSWPDDERLFYKAIGWFFFWSFL
VVEIFHVAYIVKHFRDISDAVLTGTTVTALLEALVRLYIILTKRSIINHILLKIWKQFWNVNV
VINRITRNQLKKKARVSTILTSIFLVSSIICSIKITSDAFLQNRGMVLKSVFPFDSTKPFSYELI
YIIHYCTVWCGLFVINAFDFFFVALVTNCSIQFAILQDAFKNILTGTSKGQRVAIFGQKHSNI
SDKDMLLKCLEQHQILIGICNELEESFNISILIQFVVSISAICAASLILKVDSNQFLKMVMYA
AAHLAQLFYYCFAGHGLSYESDKLSDAIYGCNWHLFYDRDFRKALVLIIQRSQRVQYLTA
AGIAKLDFASFIKVMRLSFSFYTLLNSLLAKNI

>AcorOR42
YNAFDLERRILWFYGIYFSKDFRPQKLHYLRVIATSFVINTLVLGIVMEMIVDHNNLETVFQ
SIYYIIVVIIGQIKTFSLYRSLSQFNSLEDMLQDVIFNAEISTGCTSISKAVDTYKIFKRIFWGT
ASFCASVYSLMPLLSGDLSIPIWYPSESFKLYCQLFEIVCIWTIAASLLSIDVIIMGLIYFMSA
QINTLNYNLRNATDRNPDYDAEKQEKQVQDNLRICIRHHLAISEFVSKLEEIFKFGLLLQIF
SSIIAISSGGIYVVVVPLTPSSYLLLGTSMSVVLLQIAMYCWAGQGLITESDQIGESCYMSE
WYTCNTATRKMFFIIMERSKR

>AcorGR1
RSDLDTTLLSMTTDIMQVTTSTLQIVVSWFMSAVSQEKTMSFLKRISDVDKTFRQLGVYIY
YDAVHKSVIQRLLIRMSLVLASTISQLFLYQYQWNVGMISFYVTYYFPILINVLIVEEFYIYT
NLLRTRYEILNQHLIEVQKYNDSCKEKISYIKLTGVIGSKLSTLRIICPIHHELTKIAKLLNEA
FGVILLMSFQSSFVTIIVSLYDCSVLLQYFNIEHIRELCAAIIMCCTYVLDCLYICYSCHSTVE
SANKSGRLLHQIDTDDVDVKDQIEMFSLQIVNEKLEFTAAGFFTINYGLLFSIIGGITTYLIIL
IQFSADPNESKH

>AcorGR2

MLFSRKSKDHLVRCNVLTYWQSTLSGLIQPRNVYDSLIPLHLITKCVGLSPFNYNILNEKPL
YQTSLLGSSYSFAIMILFVGYYIYAVEERDESTDSNKVARSIDMYHLYGSIIVMSACIILNSY
HQKTLIQAVKSLNEADLNMAGYSSKISWKKSRNLIFGYFSITLAVLITCEMLNCTMFLRQV
GTLTTYCLLMCYIPMVINSFAEAQFVSYILLLKQRFAILNDELRSLITQKKYLPTVKITKVGP
VINREENRPKPIAKSLICVRQMHSQLCEIGALLNKSFSLQILLNTGDVFIGFTTLAYYCFDG
CMKLYLNEDGSNLYNTVTTGVWTLVKLSRLLTLTLSCSIVKNEAHLAGHIIYKIDNRYESE
LSSEIYSFGKQIIHWNLKFTAFNFFDVDMSLFYAVVSSATTYLMILLQLDIANKQIEKSMEIN
RL

>AcorGR3
MDYGNNTPIHCARNPHQRKIEGNVKAQRLSDAGLENSAKGDSEPDPELLEQFDSFYQTTK
SLLVLFQIMGVMPIERSAKGITTFRWFSGATIYAYSLFVAETIFVTIIFKERLLLVLQKGKRFD
EYIYSIIFLSILIPHFLLPIAAWTNGHEVAHFKNMWTHFQLKYYQVTGTAIVFHNLTLISYSL
CIFSWVLGVAIMLAQYYLQPDMQLWHTFAYYHILAMLNSLCSLWFINCTAKGRVAEDLAQ
NLHNALESPDPASRLAEYRDLWVDLSHMMQQFGKAYSGMYGMYCILILLTTIVAFYGCLT
EILDHGLSFKEAGLFLIAFYCMCLLYIICNEAHYTTARMGPEFRERLLSVNLMAVDSRTRQ
EVHMFLTAIDKNPPTMNLNQYADINRRLISSTVTSMATYLVMLMQFRSTLMRNAAIAAKR
SAMNLNRTGTNATT

>AcorGR4
MYRPTDVNNLSFLGSNNNYKPKKSVYLEGASVFYQKNKVTQVAPALSNNAQWNNLPYS
ASEGGVVQECLKPIIMLERSMGIFPISVVPGGFSKVTLPWMIYSVFVFLLILSYIGYIKWDK
VEIVRSTEGKFEEAVIDYLFTVYLIPVVIIPIAWYESSRMASVFSEWMAFERIYQTITHKKLP
LFMGNKPLLVTLGLPILSCGTMVVTHITMVHFRIVQVVPYCFINAITYIVGGMWYLHCDLI
GRVATVIANDFETALHHIGPSVRVAEYRSLWMMLGKLTRNVGLGSCYVITFLCLYLFLIITL
TIYGLLSQIQDGLGVKDVGLTITAFCAIGILYFVCDEAHYASNCVRVYSIRMRCLTKAIYYL

>AcorGR5
MNRTIMSGEIFIKYPKAATIFAAICAILFCIVGFLGNFVTILALARCPKLISQATTAFVLSLCIS
DLIFCSVSLPLIASRYIYERWILGMTCCKLFPVLLYGNVALSLLNMVAITINRYTIISYYPYYS
KLYSKISILVQLVFIWAGSFLIMLPPLLGIWGQLGLHPPTFSCTILEKNGKSPKKFIFLVGFAL
PCVVIIISYICIYLKVKKSTKKLRKHQVNDSTRNKNSRREREDGRLTKLMLLIFVCFVFCFL
PLMCVNVFDDEVRYPTLHVFASILAWASSVVNPFIYAASNRQYRSAYSKLFNVFRSSMTAT
DSRQLSNSHKSRGTDNKNNHQLAKVVPAE

>AcorGR6
PTIVNTTIKLQFHLYNLILITRTEIINANLNDRIASSTPASKFTAFYQMEKDIESTMKIHKKIT
DTSRLVNRIYGFQELFSFALCFVVLLSEGYIVLYSLTVGEGEAENFGYTLFSSLRLVIFYLLQ
LLVDLRACMLLCAKINHTKNILFKIKVEPENEESRNVVMVAVFKLMHDKLEMTACDLFN
MDFSFVFSMFASITTYLLILLQFDIDAAKNRRANPLVNSSTA

>AcorGR7
NNRIRTAAQTKLILWAEVRRYYNKLYELSFRVEEKISPIILISFTSNLYFILVQLHGSVKKRNS
AMESVYFFFSFGLLCMRTLAVCLFVGNVDEESKTSIKLL

>AcorGR8
SFQFYCNFILSLLIRYFVLLINERFHQLNLMIEHHPEEVINTEGDQKKNVYVANSKDLVKAY
HLLLEQVNISNNVFGIPMLFLSFCFIVDILNMLLMWIVYSFQDNAQMKGVVFGIDMLILCC
FSVVKDLITYLALVSSCDQTAREAGYTSFACYKLLYELSKSNNLYDSYTSKEKLSGRYDIT
LLAIQSSNVSVCFSAAGFFVMDFSTFFTLISFIMSYLVVLIQFNDRL

>AcorIR21a
QADITDSEMKLRSVIITIFTINIAMAKLEKRALQKAHERSRIDKLMDKFLPRQDYDKTTSL
AELFIQIFHDYLSECVPIIIYDDKINQYYPLLDIVFQKVNISFIHSMVVVKNGKNVDRNFTYT
PDTHCFNYILLVDDIFSSKYLIGKQSTPKIVLITNSSQWRVNEFLMGDFARNLVNLLVIAPST
SPSITETDICYILYTHDLFVDGLGSSVPKVLTSWRDGELKRRHVDLFASKMKKGFSGHRFIT
SVTHEPPYVIQRGFDENDGIVWDGIEIRLLTLLSQLYNFTIDIKNFKDENFKSPTDKIIDNINN
GIVNVGLAGLYLTVDRLEVGDISYPHSYDCAAFISLTSTALPRYRAIMGPFNWTVWLCLTI
VYLMVIFPLALADKLTIKHLLKNPEEMENMFWYVFGTFTNCFTFGKDTWTKSRKLTPRVL
IGFYWLFTTIITACYTGSIIAFITLPLYPQTVDTVAQLLAGRFRIGTLDKGGWEYWFQNVSD
PQSQKLVKYIEYLPDIESGLKNITRAFFWPYAFLGSRARLNYIVQTNFTTTSKRSLFHISTEC
FAPFGVGIIFAKKSIYKNTIDKGISYLQQAGIVSKFESDIRWDYMRSPTGKLLQASGSTLKM
LTVDDRSLALDDTQGMFLLLGAGFLLGFFALISETLGGCFRCLKRKRCDSSISSIPSNPRLYS
LPTPRESIDSIQFSNNIQWDFAGEKMKTNINDNSRIDLHNFEKFFGEHINLIHYRRVSDRVNI
AR

>AcorIR41a
MYLQINILANFIINKYFNNNNCLLIITDRNNNFEYNGNLSYVYVKLNGIEIPYNLVFRSYGC
QGIVITCQSPVSIFENLELGMKLGSDRFNYRKYLFLTVTNQLETSLDVLKSKAAEFVADILI
VAVDSDESIFDLYTHKFAGPKEKSTDITWLDRWYSINNSFLFDSNLYPDKLENLEGRPFRIIC
FTYKPYCIIDPPDGTDMLVALEYARKHNMTPELVVDEAGEWGNLYDNWTGNGVVGNLA
QDMGDIGLGALYTWEREYSFFDYSKPTMRSGITCIAPAPRLASGLATPFVSFSMELWIMTL
SSYFLASVALLIVLSVTISDENDKKRNVNNIMLSLSLAGRIFLLQSFQKVPNLEQSRITFGLA
LILSLMLNTIYSSGLSSTMTIPRYYGTIHDADDLAASEIKWGATSTAWIESIDSDSRKVFVHI
VKNFQILTEKELAAFNDEDLAYAVEHLQGGNLALGSYISLDGIQRRRLLDEDLYWEYCVL
MLRKNSIFLSSLNDVILAVTESGLLYYWEHQTVYKYMDMNMQKAVKMSLRANNPGGSN
SIVKLNLDHVIGAFTIWGVGILISIIVFICELIKNKYNRGDNKV

>AcorIR75q
FETMISWKYNTLLFTALCITVICSENMEFIEIFKDFLLMQKRPTKVFLYLCWPVKEKVLVAR
YLNKYSFSFQFNTQLKLPIFKKSTEQLFLLDIRCTQYLNLLYMANLNKLYQQPYRWFLIVN
ESITLPHFLEILVDSQLYIVQNASPAVYVISSLYKISKYSENFVENDVARWSRSLRFSYFHPVS
AVRNRTNLLGMPMNISYVITNNDSLNHLWDYRDKHIDGVSKLNYILSHYIIDAINVSGYFI
VRSTWGYKNVTTNLYDGLIGDLQSGLAEFAGTASFFTPDRLLIVDYIAPTTPTRAKFIFRAP
PLSYVRNVFTQPFDKMVWYASFLLLAVISIIVYVIVKWEWSSSKFQNISTKQNVMKPKYV
DVLLMELSAICQQGSETEPRSGAGQISVIVVFLTFMFLYTSYSANIVALLQSTSDSIQTVDDL
LNSRIKLGVEDKPYSYYYFKIQTEKTRKAIYTQKVAPVGQKPNFMNVEEGIRRMKDEFFA
FHVECASGYKIVADIFQESEKCGLREIEYWQIIDPWMAVKKNSSYKELVKVAYRKLHESGI

QNREYRRLYAKKPICQSRGSNFISVGFIDCYFPFLIFGVGNLITIAVLFIEILYKSRKRFQQKIY
I

>AcorIR75x
MLLTCLDFGLSITKHNSIINFLSSYAKSKNVMLTLHTCWNKDMFLDKLLDFSKIANYGNDY
ATFVSTHTHIFQKYSFNNLLIIIDLNNCPKAWSSLLQMNQTDSFAAPAKYLIFIDNIADIESTI
RNFTIYPSSDILIAEKHASDYKLHGIYRINAISDLIWENYGYWSCEHGLVEYLKKYPPSQRR
HDLKQQNLKVMIRITNNDTWNHLEDFRYTSVDGFTKLSYGASKCLFEYCNANVTYLGTE
FFGYKDKTGNYNGIVGALMRGEIDTSGSPMFTRIERVPLITYMTMQSPYYIKFILKKPPLSY
VKNIFFSAFDYKVWAAVIVSFVVLVIVSLFIYNTEAKQAKINFQSKLTISDVILVSLEVLSQQ
GTYMDFQRMSSRILILTFLVAFFFIFIAYSGNIVAMLQAQVELKSTKELVDSRLDLGAEDINF
MQLFLSQDTDAVGKQVYQKIGENGYYPLAVGMEKVRKGFFAYHAELAEAYYYMREKYT
NNEMCCLQEVEGYFQYLRGYSVTRKRSPYKEIFKTGLLKVDEYGLKLRHYNLWYIKPICH
TKGSNVGSVGLIECRMAFFLLIYGTLISMLFLLAERIIHYTQTTL

>AcorIRx
KNHRLPFTIKDAAIRHDDRNGLQLSHKTCNLLARGVHTIIGPNSYPMSRHVGVICSGKDIP
QVLTRSYSSDEDYNNFAINLHPHPPILEKLFVELLNKLQWTKFMIIYQSNQDLVKVHQLLS
YKSNIYDIKLSQLILDDRMSYRLMLNAIKKSGECHFVVVCDLLTLKRFLQQAQQVGLLTE
KHHYVIYNFDMSNIDVEPYQYGGCEIISVRFFDPYSTEIQDAFNAVDEELYSNYGIETDGQT
LNLETALIMDAVKLIHTTLKEHMLPEHIDNQPLHCNDSEAWWHGPSLRNYLNVANVKGY
TGLIKFDPKGYRSDFEADIIELKSEGLTKIGTWNTTDGLIIERPEKKDPLPDDLSDVRGRTLN
VVSALTRPYGLMKQATTRLYGNDQYEGYGIDLIHELSKELGFQYKIIPQEDGVNGSKDNK
TGKWDGMIGKVMSGEADLAIGDLTITSERENAVDFTLPFMTLGITILYKKAEPVPPSLFMF
TSPFSPQVWLLLIVAWIFVSLSLFVMGRLSPSEWQNPYPCIEEPEYLINQFTFKNSFWFTVG
SLMQQGTELAPVGISTRMLAGVWWFFTLIMVSSYTANLAAFLTVTTLNTPFSSIDELAKQ
DEIKYGAKANGATAFFFKDSDKPVYQKIWKYMNNNPDLMVKDNMLGVNRVLKENYAFL
MESTTIEYITERYCTLAKIGELLDEKGYGIAMKKGSAYRQRFNTAILKLQETGMLTTLRMR
WWKEKLNGGACDERSSTATVTALDLQNVGGVFLVLGLGAFFGVVMAVLELSMDIMRYV
KHQKAKYKQQMREEMKFFIEFKRNVKPSRKTGQENEDAVEFPFNINYMENYINENNQNE
