# Supplementary material for: Chemosensory Gene Families in Adult Antennae of Anomala corpulenta Motschulsky (Coleoptera: Scarabaeidae: Rutelinae)
Source: PLoS One. 2015 Apr 9;10(4):e0121504. doi: 10.1371/journal.pone.0121504 (PMC4391716; doi:10.1371/journal.pone.0121504)
Supplement: S1 Table — (PDF) [file pone.0121504.s006.pdf]

S1 Table. PCR primers used for RT-PCR and qRT-PCR.

	Forward primer (5' - 3')	Reverse primer (5' - 3')
RT-PCR		
OBPs		
OBP1	GCAATCATACATCGGCTAC	ATTACTGGTTCTGCTTTCG
OBP2	TTTTGCGAGTACCTTGGTG	ACAGGCGAACAGAAGAACA
OBP3	AAACTGACGCAGGACTTACA	TTTCGTTGAATGAAGCACA
OBP4	AAAGGGAACTGGGACGAT	TTTGGCGATTTCATAAGC
OBP5	AAATAAGAGCCGCCAATA	TGATACACTAAGCAGAAGCA
OBP6	ACCCTCTTGTTTGGCTCA	GCATCCATTAGCACCACC
OBP7	AGCCAATGCTACAACGAAG	TGAGGTTAGCTCCAATCGT
OBP8	ATGCTATTGCTCACCTTGG	CATCATCGGGAACGGTATT
OBP9	TGTATCGGTTCAAGCACTTA	TTCGCCCAGTAACATTTCCA
OBP10	ATTGCGGTAGTCGTATTG	GCTCCTTTCAGGTCCTTG
OBP11	GCTTACGAGTTCGAGGAT	AGTGGGCACTGTAGTTGA
OBP12	GCTTTAGAATGCGGTTTA	TATGCTCATCTTGCTCCA
OBP13	TGATGTTTCGGTGTAGTCG	CACATCTTTCTTGGTCCTTA
OBP14	CAAAGGTCTGCTGCTCTATC	GACCCACTAACACTACAAAGC
OBP15	GCGGTAAGTTGATCTCCCT	GCTACCCTCAGCACCATTA
CSPs		
CSP1	TCGTCGTCGTAATCTTCAT	TCTACTCTTATCGTCGGTTT
CSP2	CGCACTTCAACTGTCTCAT	GAGGTTGGCGAGATAAGG
CSP3	ATTAGATTTGGCTCCTTGT	ACTTTAGCCTTTACCTTCC
CSP4	CTACTTGGCACGGAAACA	CTCTTGGTGATGGGAGCT
CSP5	TCTTGCGATATTGTCCAG	TACCGTTATTTCATCCTTAGTC
SNMP		
SNMP1	TCTACTTACTGTTATGCGTGAT	TTGTCTGAAACTGCTGGA
ORs		
ORco	TGAGGTGCCTTGTAGACATA	CATACAGCCATTGTGAGTGA
OR1	ATTGGGCTAAATGTCCTG	ATTACCTCGATGGCTTCA
OR3	TCCTAAATGGTTTGCTCGTA	GAAATCCAGATGTGGGTGC
OR4	ACTAAGGTGGCGAAAGAG	CTTGGCATAGTAATGGAGA
OR5	ACCTGGACGACATCTTTCT	TAGGCTTCTGCATTCTTAT
OR6	TTTGACCAACCCAGCATA	TATACAGAGCTTTGCCTCAT
OR7	GTCAGGTCGCCCTCGTATTA	AGTGGGTTGTCGGAGAAGAT
OR8	CCTAAAGCTGGTGGAAACC	TGGCTGGAGTGATATTGAAGTA
OR9	TCTTTGCCCTTTCCATTAG	TGGTGTTTCGGATTCTATT
OR10	GCAGTATGGTCACCGAAAGA	ATAATGGTCGCTTCATCCTCA
OR11	ATGCTTTCGGTCTGCTCTG	GCTCAATCCGCTAATCTCA
OR12	AAACTCCTCCATTTCTACCTG	GAAACAAAGGCTTCCAACG
OR13	GTCCAAGAGGCTATAAACG	TTCATTGCCACGATAACAG
OR14	AAAACGTCAACATGGGTATG	CATTTCTGCTGCGAAGTTTA
OR15	AGTATCTATCCAGTCGCTTTC	TACAACCTGCCTTAAACTCTT

OR16	TGTGCCTGCTTCTACCTTC	GCCATAATCGTTTCGCTGT
OR17	AGCGGAGTGTTTAAGGTTG	AGAAAGTCGCCCAATACG
OR18	CTGATCGCTGATTATTTGAC	TGGGCTCTACTTTATACTACC
OR19	TTTATTAGGTGCGGGAGC	ACTTCACATTCGTGTTTGC
OR20	GGTGGCAATCATTAACTCA	TTAAGGGATCAAACTGTCG
OR21	TTATTCCTCTTCGCCGTAT	GGTCTACTAACCCATCAACA
OR22	TCGTGCAATAACGTGTAGAAT	TGTCAGTCGGGAATAAAGAT
OR23	TGCTGGAACAATAAAGGG	AATACGGAGACATCTACACGA
OR24	TGGTCGTAATGACGTTCTGT	TCATAGGAATGACGACGAT
OR25	GCGTTTAATCCTTGGTTGTTAC	TGTCTTGTGAGATTTGCCAGTT
OR26	TGCTCCAATAAGATAGTTCG	ATAAAGATGGCTTCTCAACA
OR27	GTCGCCATCTTCGTTAGG	ACCGACGCACTCGTTCTG
OR28	TGACCAGCCCAGCAGTAC	TCTTGGCGTTGTCTTTGA
OR29	TATGGGTTTGCGGTTTGT	GGTTGCTGCGTATTTGAC
OR30	GGCATTGTATCCTCTGACTTT	CTTGAGAAATCGCCGTGTC
OR31	TGCCATATTTATCTCGCTTTG	TTCATTTAGCGGGTTGGAC
OR32	GTTGGATTTGACGGTTTA	CGTATGGTCCTCCTCGTAT
OR33	CTCTGAAACCCTTGCTTGT	GTTATGCGTTGTTCCTGCT
OR34	GCGAAATAGGAATACGATG	GGAACAACTGGGTGTAGTGA
OR35	CGCTCCCTCGTAGTTACAT	TTCCACCATCTCCAGACAAA
OR36	TTTGCGGAATTGAACATG	TGCGTCTAGTTGGGATGT
OR37	TCTGGACACGGATACTACATT	GTTGGAAACCTGGATCTTTAT
OR38	CTTCCAAAGCTCTTTCGTAC	ATTATCGCTGCTGTTGTTCC
OR39	TTTATTCAGGGACGCTACT	CTCTTATCAATCCACCACC
OR40	ATGGGACTTCTATGCTACGAC	TTGACGACAGCGGATGGT
OR41	ATTACACCCGTAGATGGC	AACAGCTTTATTGGAGGC
OR42	TGGTTCTTGGCATAGTAATG	AACAGTAAATACGAGGATGG
GRs		
GR1	GTAGTATCCTGGTTTATGAGCG	AAGAATCCTGCGGCTGTG
GR2	AAATCCAAAGACCACCTCG	TCCCATCCTCGTTCAAATA
GR3	CCCGAACTACTGGAGCAA	TGTGATGGTGCGTTGTTTA
GR4	ATATTTAGAAGGTGCCAGTG	AATTAGAGGCGTAATGAGC
GR5	ATGTTGGAATGCGATAGGC	CGTAGTATGCGGTGGTTGC
GR6	ATCGCATAGCAAGTTCCAC	ACCGCTACCATTACCACATT
GR7	TCCAACAAACAGGCATACT	TACGGAGATACTACAACAAGC
GR8	CTTTTGATTCGCTACTTTG	GGATTCTTTTGCTCCTTAA
IRs		
IR21a	TCTTCGTCGATGGATTGGG	CGCTGTTTGTCGCCACTT
IR41a	GATGGTACTGATATGCTGGTC	GATGAGCGTCTTGGTAGGA
IR75q	GAAGGTTCTAGTAGCGAGGTA	AAAAGCACGACATCACATT
IR75x	TCTGCTAATTCTGCGTGAT	ATTACGGTTACTGGTCTTG
IRx	CACCCTCATCCGCCAATA	CGTTCATCACATGCTCCC
qRT-PCR		

OBP1	CGTTGGCGATGATGGTGTAGTAG	GCTCCAGGTTTGACTCCACATTT
OBP3	CGTTGAATGAAGCACAAATGAAAGC	TGAAGAAGGCATAAGATAGGTCACA
OBP4	TGGCGTGTATATCGTGGCGTTA	CAGTGTAAGTAGCACCTAGATGTATGG
OBP5	GGATAGCGGCGTTGTGAAGAATA	GCACGGTTCAAGAGCCTGTT
OBP6	TCACGCTGATGCCGAATATGAA	TGTAGGACCGCTGTTGTCTTCT
OBP7	GGAATCCAAGATGCCAACGGTAA	ATGATCCTTAGCGAGATTGTGATATGG
OBP8	ATGCTATTGCTCACCTTGGAACTG	CGACCACCAACAATACATGCTTCA
OBP9	GATTATTGTTCTTGCTGTGCTGTGT	GCTTCGGATCGTCGCCAAAT
OBP10	GGTAGTCGTATTGTTATTCGCATCTTC	TTCGCCAGCATCCGCCTTAT
OBP11	TTACGAGTTCGAGGATCAGGCTTAT	ATTAGTTCTCCATCGGCAGTCAGA
OBP12	GAATGCGGTTTATCTTCAAGCCAAA	TCGGTGATCTCAGTGTTGTTCCTA
OBP13	CCTATGCTTGATGAGGTCACTACAA	CGGAGGATGGTCATGCTTCTTG
OBP15	GCTGACAGTCTTGGCGTATCTC	ACCAGTATCACATTCATCGCTACC
CSP1	CGCACTTATTACAATACCACCGAGAT	GTCGTCGTAATCTTCATCTTCGTCTAA
CSP2	CGCAGGTATCTGAAGAAGCTATCG	CGTAACCTAAGACCTTCTGGATTTGT
CSP3	TGTTATCCTAATAGTTCCGATCTCTGG	CGAGTCCATCCACAGTACAAGG
CSP4	ACGACGACGACGACGACAA	GTATTAAGGACAGCATCCGCAGTAT
CSP5	TCGTGTTGTGCTTAGTTGTAGTGG	GATGTTGTATTGAGCCTCGTACTTCT
SNMP1	AGACAGGAATGGTGGTGGAGTG	AAGTGAGGCATCGTAGCAACAAC
ORco	GCTGAACTATTACGCTGAGACTACC	ACTTTGTTGGACGAGATTACCGAAA
OR1	CCGCATACATCGTGGTTTCCTTT	CAACTCCTTGAAGTCTTAATAGCCTGT
OR2	ACTCTTATTGTGCCTCTCACTGTT	AGAACCATATAGTACGATCCTGAAGC
OR3	ATCAACGCTTAATCCGCTCAGTG	GACCTCGATAATTCATTGCTCTTCCA
OR4	CTGATTATGATGCTGGAACACAAGAG	CCATTCGGACATATAACAAGATTCACC
OR5	GAATCTGCTGCGAATCTCAACGAT	TGCGATAACGGAACCATACGATAAC
OR6	TTGGTATCCTGCTGACTGTCCTAA	CGATTGTCCTCACCATTCCATTGT
OR7	GGTGAACTGGAACTTAACCAACTCATA	ACGCACGCAATATCGCAACAA
OR8	GCTGGAGTGATATTGAAGTATGATGGT	GTGTTCTCAAGTTCTTCGCCGTAG
OR9	TTCACCAAACTTCCACGCAGAG	GCTTCCTCATCGCCTTATTCAGAA
OR10	CTGGTTCAAGCATTAGGCACGATA	GGTCGCTTCATCCTCATCATTGTAA
OR11	CTCAATCCGCTAATCTCAACCAGAA	TTGTGAAGAGACTTGAAGCAACTGT
OR12	TGTACGATGCTCTAAGTAATCCAGGTT	GCTTACTGTTGAATGGTAACGCTCTTA
OR13	CAGATCCTCACAGAAGTGGAGATATAGA	CCTGCTCGTTGTCGTTGAACC
OR14	CCACCTACATTGATGGATGAGATGC	GAACTAGAGATATTGCCGCTACTAACAG
OR15	TGTGTATGACCATGTTCCAACTACTC	TATGCTGCTACTGGCACTTGAGA
OR16	CCTGCTTCTACCTTCTTACCGAATATAC	CGCCTGTTGTCATCATCACTACG
OR17	GATCTTCACGAGTGTAGGAGCAATT	TACCAAGTTGATTCGTAGCAAGCAT
OR18	CAAGGCGAGGAATCCGAGGTT	GCCATTAGCACTGTTAGGAAGAGAC
OR19	ACTGTAATCGTAGAATAAGAGGAGTCC	GGTAAGGCATTATGTTCTTCGCTAA
OR24	TTATGCCGCCATTGGAGTGTT	GTATATCGTCGTCATTCCTATGAGATC
OR25	GTCGTGCCTAACATTGCTGATTATAC	TTACAAGTGCTGCTGTGTCGTT
OR26	GCCTGTTATATCCTCACTAGCAATCC	GACGAGAGTGCTCCAATAAGATAGTTC
OR27	AATACAGAAGATACCGACGCACTC	GCTAGATATTCAGTCACGATGATTACC
OR28	GCCAAGATTAAGTCACGGTTGAGA	CGATGTGTAACAAGCAGTCGTTATATG

OR29	CGCATCGTTAGTAATACCTATGTGGAT	CCAGCATTCCTTGAACAGTAGAGAC
OR30	TCAGCATAACCATTCTACTCCAATTCG	TCGCAGCAGTCAGGCATTGTA
GR4	CTAATTGGACGAGTTGCTACAGTTATC	CAGGACCCTAAACCCACATTGC
GR5	TAATGCTACCACCACTACTCGGAATA	ATTAACGCACATCAATGGCAAGAAG
GR6	GGTGAAGGTGAAGCCGAGAAC	TGGTTAATCTTCGCACATAGTAGCA
GR8	GTGTAGTGAAGGACCTGATAACTTACC	CACCGAAACATTTGAAGACTGTATAGC
IR21a	CAATGGCGAGTCAACGAGTTCTTA	CCAGACAATTCCGTCATTCTCATCA
IR75q	AACGAGTCAATTACCTTACCACACT	CGCAAGCTCCGAGACCATCT
IR75x	ACGCAGAATTAGCAGAAGCCTAC	GGTAACGGAGTATCCTCGCAGAT
IRx	ACGCAGTTGACGAAGAGTTGTATAG	AAGAGAAGGACCATGCCACCAA
G3PDH	TTTCGTCGTACCGCATCAA	ATTCTCGTCAGCAACTTCACC
