# Supplementary material for: Chemosensory Gene Families in Adult Antennae of Anomala corpulenta Motschulsky (Coleoptera: Scarabaeidae: Rutelinae)
Source: PLoS One. 2015 Apr 9;10(4):e0121504. doi: 10.1371/journal.pone.0121504 (PMC4391716; doi:10.1371/journal.pone.0121504)
Supplement: S2 Table — (PDF) [file pone.0121504.s007.pdf]

S2 Table. Percentage of homologous hits of the *A. corpulenta* contigs to other insect species.

No.(female)	species	Percentage (%)	No.(male)	species	Percentage (%)
40961	*Tribolium castaneum*	44.4	37428	*Tribolium castaneum*	44.6
5127	*Acyrthosiphon pisum*	5.6	4284	*Acyrthosiphon pisum*	5.1
2907	*Camponotus floridanus*	3.1	2530	*Camponotus floridanus*	3.0
2813	*Hydra magnipapillata*	3.0	2378	*Hydra magnipapillata*	2.8
2730	*Harpegnathos saltator*	3.0	2037	*Harpegnathos saltator*	2.4
1496	*Danaus plexippus*	1.6	1284	*Plasmodium falciparum 3D7*	1.5
1374	*Aedes aegypti*	1.5	1249	*Danaus plexippus*	1.5
1102	*Pediculus humanus corporis*	1.2	1132	*Aedes aegypti*	1.3
1025	*Bombyx mori*	1.1	961	*Megachile rotundata*	1.1
994	*Nasonia vitripennis*	1.1	958	*Pediculus humanus corporis*	1.1
31824	others	34.5	29758	others	35.4
