# Supplementary material for: Chemosensory Gene Families in Adult Antennae of Anomala corpulenta Motschulsky (Coleoptera: Scarabaeidae: Rutelinae)
Source: PLoS One. 2015 Apr 9;10(4):e0121504. doi: 10.1371/journal.pone.0121504 (PMC4391716; doi:10.1371/journal.pone.0121504)
Supplement: S3 Table — (PDF) [file pone.0121504.s008.pdf]

S3 Table. Comparison of the number of chemosensory genes identified in different coleopteran species.

Species	No.						Reference
	OBPs	CSPs	SNMPs	ORs	GRs	IRs	
Ips typographus	15	6	3	43	6	7	[33]
Dendroctonus ponderosae	31	11	3	49	2	15	[33]
Tribolium castaneum	49	20	2	341	220	10	[25,45,46,47]
Anomola corpulenta	15	5	1	43	8	5	-
